# Synthesis, Biological Activity, and Molecular Modelling Studies of Naphthoquinone Derivatives as Promising Anticancer Candidates Targeting COX-2

**DOI:** 10.3390/ph15050541

**Published:** 2022-04-27

**Authors:** Povilas Kavaliauskas, Felipe Stambuk Opazo, Waldo Acevedo, Ruta Petraitiene, Birutė Grybaitė, Kazimieras Anusevičius, Vytautas Mickevičius, Sergey Belyakov, Vidmantas Petraitis

**Affiliations:** 1 Department of Organic Chemistry, Kaunas University of Technology, Radvilenu Rd. 19, LT-50254 Kaunas, Lithuania; birute.grybaite@ktu.lt (B.G.); kazimieras.anusevicius@ktu.lt (K.A.); vytautas.mickevicius@ktu.lt (V.M.); 2 Joan and Sanford I. Weill Department of Medicine, Weill Cornell University, 1300 York Avenue, New York, NY 10065, USA; rop2016@med.cornell.edu (R.P.); vip2007@med.cornell.edu (V.P.); 3 Department of Microbiology and Immunology, University of Maryland School of Medicine, 655 W. Baltimore Street, Baltimore, MD 21201, USA; 4 Institute of Infectious Diseases and Pathogenic Microbiology, Birstono Str. 38A, LT-59116 Prienai, Lithuania; 5 Biological Research Center, Lithuanian University of Health Sciences, Tilzes Str. 18, LT-47181 Kaunas, Lithuania; 6 Instituto de Biología, Facultad de Ciencias, Pontificia Universidad Católica de Valparaíso, Valparaíso. Av. Universidad N° 330, Curauma, Valparaiso 2373223, Chile; felipe.stambuk.o@mail.pucv.cl; 7 Instituto de Química, Facultad de Ciencias, Pontificia Universidad Católica de Valparaíso, Valparaíso. Av. Universidad N° 330, Curauma, Valparaiso 2373223, Chile; waldo.acevedo@pucv.cl; 8 Latvian Institute of Organic Synthesis, Laboratory of Physical Organic Chemistry, Aizkraukles 21, LV-1006 Riga, Latvia; serg@osi.lv

**Keywords:** non-small cell lung cancer, naphthoquinone, ROS, mitochondrial damage, anticancer

## Abstract

Non-small cell lung cancer (NSCLC) remains a leading cause of cancer-associated mortalities worldwide. Therefore, it is crucial to develop a novel therapeutic option targeting localized and metastatic NSCLC. In this paper, we describe the synthesis and biological activity characterization of naphthoquinone derivatives bearing selective anticancer activity to NSCLC via a COX-2 mediated pathway. The biological evaluation of compounds **9**–**16** showed promising structure-dependent anticancer activity on A549 cells in 2D and 3D models. Compounds were able to significantly (*p* < 0.05) reduce the A549 viability after 24 h of treatment in comparison to treated control. Compounds **9** and **16** bearing phenylamino and 4-hydroxyphenylamino substituents demonstrated the most promising anticancer activity and were able to induce mitochondrial damage and ROS formation. Furthermore, most promising compounds showed significantly lower cytotoxicity to non-cancerous Vero cells. The in silico ADMET properties revealed promising drug-like properties of compounds **9** and **16**. Both compounds demonstrated favorable predicted GI absorption values, while only **16** was predicted to be permeable through the blood–brain barrier. Molecular modeling studies identified that compound **16** is able to interact with COX-2 in arachidonic acid site. Further studies are needed to better understand the safety and in vivo efficacy of compounds **9** and **16**.

## 1. Introduction

Non-small cell lung cancer (NSCLC) is one of the leading causes of malignancy-associated deaths worldwide [1]. Cigarette smoking and exposure to various environmental pollutants results in rapidly rising cases of NSCLC worldwide [1,2]. Approximately 60–70% of all patients with NSCLC present with late and advanced stages of the disease involving multiple organ systems [2,3,4]. Patients with advanced-stage NSCLC are generally treated with aggressive surgery, chemotherapy, radiation, or a combination of these treatments [2,3,4]. Despite aggressive treatment, the five-year survival rates among patients with late-stage NSCLC remain poor [2,5].

Numerous therapeutic options targeting NSCLC have been recently developed [2,3,4,5]. Classical chemotherapy drugs, such as cisplatin, doxorubicin, and paclitaxel, are often used for the treatment and control of lung cancer [6,7,8,9]. These drugs target various cell cycle stages leading to the induction of apoptosis in cancerous cells, resulting in the full remission or control of metastatic spread. On the other hand, many classical chemotherapy drugs are often associated with great and potentially fatal systemic toxicity and multiple off-target effects [10,11,12,13]. Thus, it is important to develop novel compounds with selective activity, good tolerance, and low toxicity towards non-cancerous cells.

Naphthoquinone nuclei have been previously extensively explored by the field of medicinal chemistry due to their profound and wide range of biological activity. Various natural and synthetic naphthoquinone derivatives demonstrate good antimicrobial, anti-inflammatory, neuroprotective, and cytotoxic activity [14,15,16,17,18].

1,4-naphthoquinone core-containing compounds are able to induce cancerous cell death by targeting multiple molecular pathways. Previous studies have shown that 1,4-naphthoquinones can selectively inhibit NAD[*P*]H-quinone oxidoreductase (NQO1), STAT3, and NF-κB signaling pathways or act as regulators of tumor suppressor factor p53. Furthermore, these compounds can induce the apoptosis of cancerous cells by directly interacting with DNA, leading to DNA damage and the release of ROS [19,20,21].

Since cancerous cells exhibit an increased production of ATP due to upregulated cellular glycolysis processes, better known as the Warburg effect, mitochondria-directed anticancer strategy was widely explored [22,23,24]. Glycolysis-based ATP production in cancerous cells is less effective than ATP generation during classical mitochondrial respiration. Therefore, cancer cells can survive competition with other cells and eventually gain the advantage, leading to multidrug resistance [23,24,25,26,27]. Pharmacologically increased ROS production in tumors leads to oxidative damage, ATP starvation and cell death caused by mitochondrial injury. Naphthoquinones’ structural similarity to ubiquinone, a key element in mitochondrial respiration processes, suggests that naphthoquinones could be potentially explored as mitochondria-targeting compounds [28].

Naphthoquinones were previously explored as a potential anti-inflammatory candidate. Plumbagin, a cytotoxic naphthoquinone derived from medicinal plant *Plumbago zeylanica*, was shown to be able to inhibit cyclooxygenase-2 (COX-2) expression [29]. Interestingly, COX-2 is highly expressed in transformed cells, and the pharmacological inhibition of COX-2 in various tumors leads to cell death [30,31]. Furthermore, several studies have shown that COX-2 is also associated with cellular metastasis processes in solid tumors. Therefore, naphthoquinones with good COX-2-inhibitory activity could be potentially explored as anticancer candidates.

As a continuation of our interest in the development of novel compounds targeting NSCLC, in this paper, we describe the synthesis of naphthoquinone derivatives **9**–**16** bearing aromatic moieties and their in vitro anticancer activity characterization using well-established A549 NSCLC 2D and 3D models. The obtained compounds were fully characterized, including the determination of the structure by single crystal X-ray diffraction analysis. The most promising naphthoquinone derivatives, **9** and **16**, exhibited good anticancer activity in 2D and 3D tissue culture models by inducing mitochondrial damage and causing profound ROS release via a COX-2 mediated pathway.

## 2. Results

**Scheme 1:**
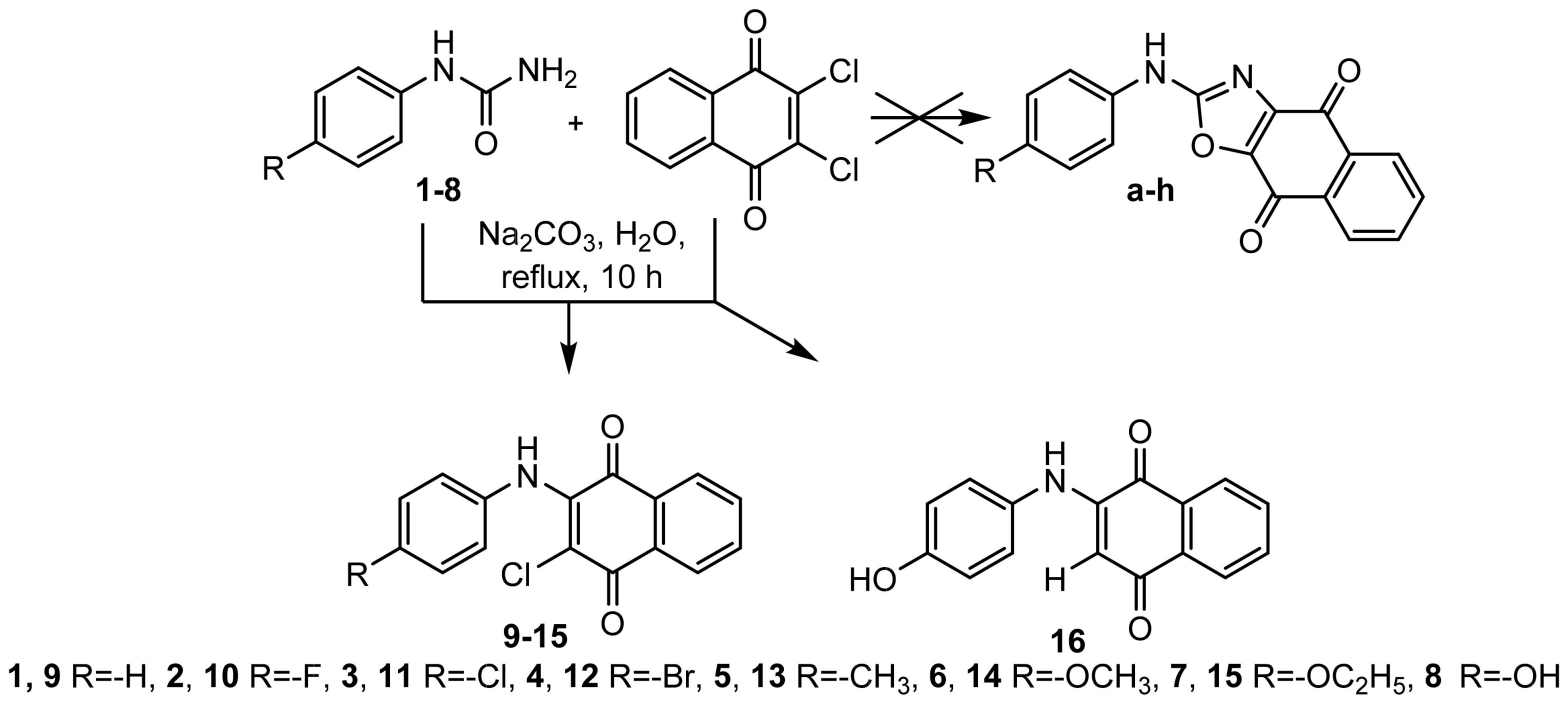
The synthetic pathway to generate naphthoquinone derivatives **9**–**16.** Compounds **9**–**16** are synthesized from the corresponding aromatic amines and 2,3-dichloronaphthoquinone [1,4,19,20,21]. By using aryl urea in this reaction instead of an aromatic amine, we hoped to form an oxazole ring, but we failed to form 2-chloro-3-(phenylamino)-1,4-naphthoquinones **9**–**15** or 2-(4-hydroxyphenylamino)-1,4-naphthoquinone **16**.

As a continuation of our interest in developing nitrogen-containing heterocyclic molecules possessing biological activity, we expected oxazole derivatives **a**–**h** to be synthesized, but this synthesis led to unexpected products **9**–**16** [26,27] (Scheme 1). These compounds were further subjected to structural and chemical analysis studies to confirm the identity of the products (Scheme 1). We then further characterized the anticancer activity using the well-established A549 cell line as a model of NSCLC and performed molecular docking experiments to predict the target of the compounds.

### 2.1. X-ray Crystallographic Study

**Figure 1:**
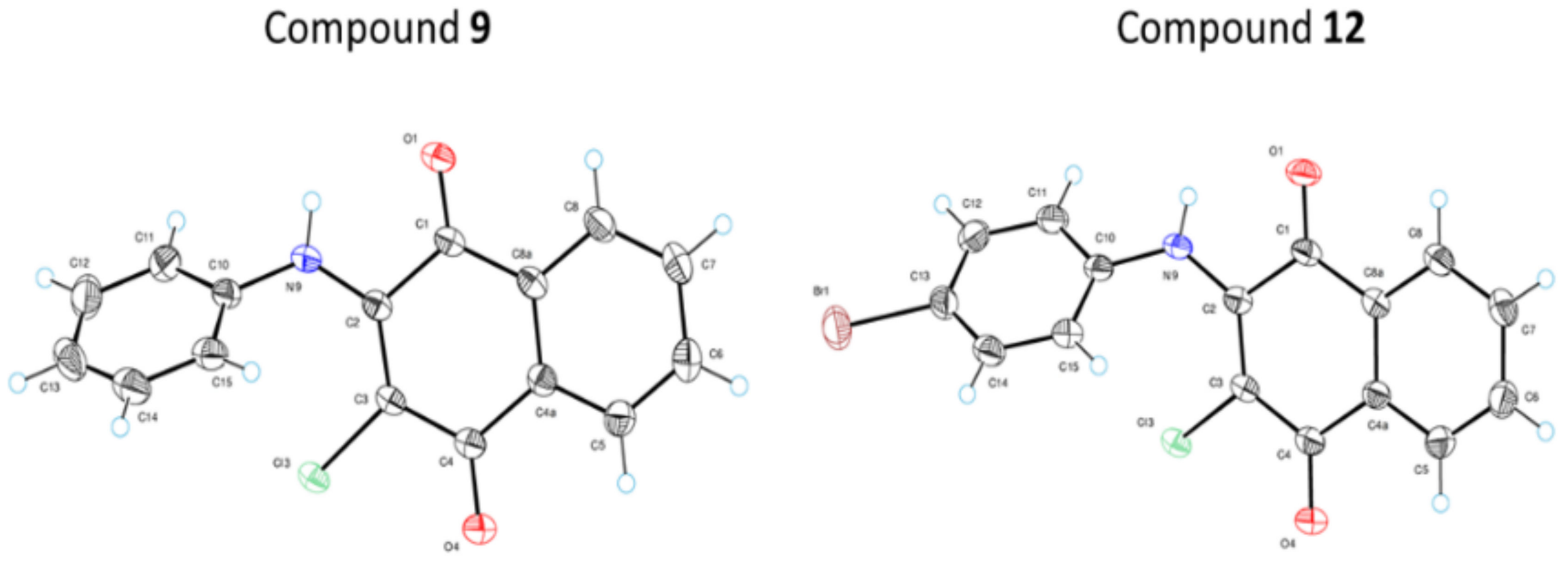
ORTEP diagram of molecules **9** and **12** showing the numbering scheme used in this work.

To confirm the structure of synthesized compounds, we performed an X-ray diffraction analysis on compounds **9** and **12**. Figure 1 illustrates ORTEP diagrams of molecules **9** and **12** showing the labeling scheme followed in the text and thermal displacement ellipsoids at the 50% probability level for non-hydrogen atoms.

The values of dihedral angles between the phenyl ring and 1,4-naphthoquinone system are 54.2(2) and 49.7(4)° for **9** and **12**, respectively. High values of these angles lead to breach of conjugation in molecules of **9** and **12**. In the crystal structure of **9** there is a moderate intermolecular hydrogen bond of NH···O type between an NH-group and oxygen atom O4. The length of this bond is equal to 3.026(1) Å (N9-H···O4 = 147(1)°, H···O4 = 2.31(2) Å). In the crystal structure, molecular chains are formed along crystallographic direction (001) by means of this bond. In the crystal structure of **12**, there is an analogous hydrogen bond with a length of 3.039(3) Å (N9-H···O4 = 144(3)°, H···O4 = 2.36(4) Å). The molecular chains formed by this bond stretch along direction (100).

### 2.2. Naphthoquinone Derivatives **9**–**16** Exert Selective Anticancer Activity on A549 Cells

After performing chemical and crystallographic analysis to confirm the structures of the compounds, we used a well-established A549 human lung carcinoma cell model. A549 cells were originally derived from a Caucasian NSCLC patient and are used as a gold standard for screening compounds active against NSCLC. By using this model, we evaluated the in vitro anticancer activity of compounds **9**–**16** and compared it with the standard chemotherapeutic agent doxorubicin. We further confirmed the selectivity of the compounds by using non-cancerous Vero African monkey kidney cells.

**Figure 2:**
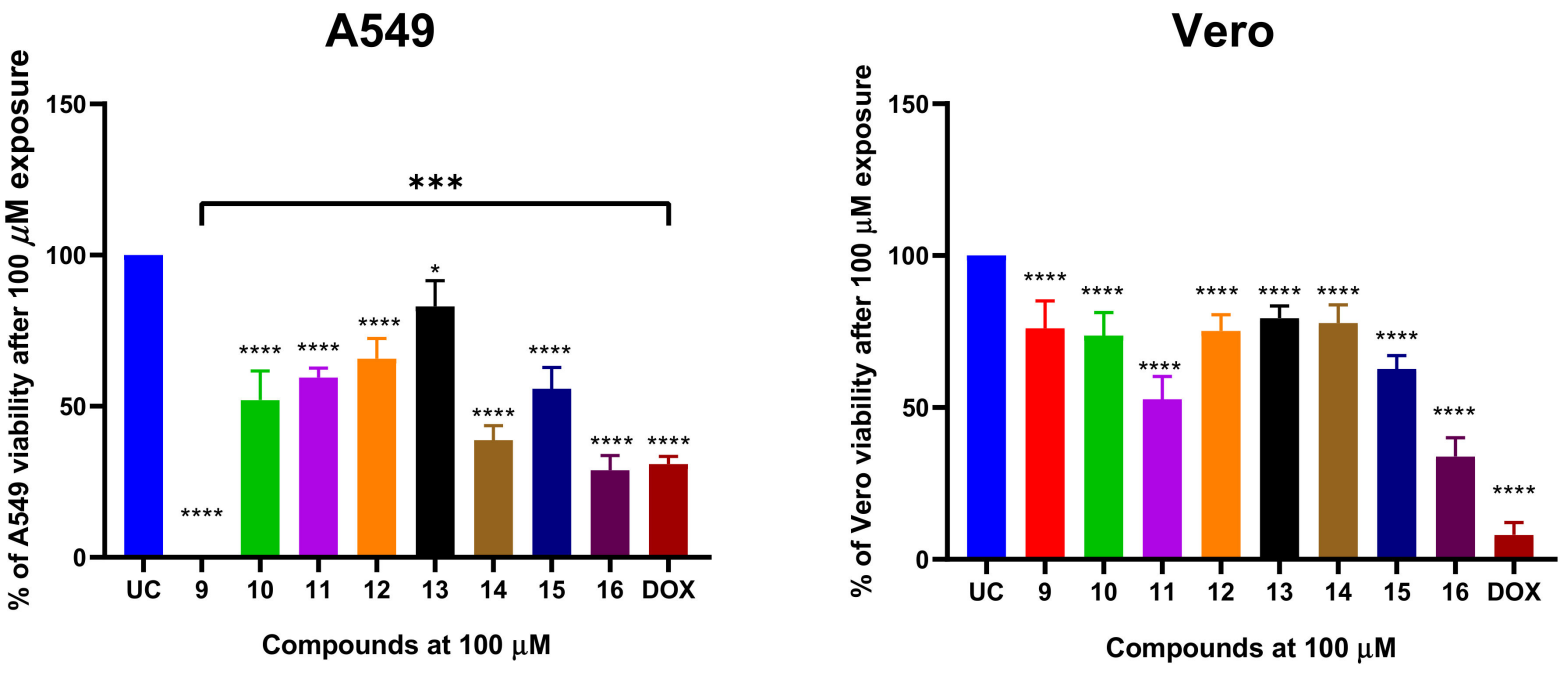
The viability of A549 non-small cell lung cancer (NSCLC) cells after 24 h treatment with compounds **9**–**16** and reference drug doxorubicin (DOX) with a fixed concentration of 100 µM. The A549 cells were treated with the corresponding compounds or comparator drug and the post-treatment cell viability was evaluated using MTT assay. The percent of viability was calculated from an untreated control. Data shown are mean ± SD values from three separate experiments for each group. The significance of the data was determined using a one-way ANOVA test. * *p* < 0.05, *** *p* < 0.0002, **** *p* < 0.0001.

We first exposed the cells using a fixed concertation of 100 µM of each compound or DOX for 24 h and evaluated post-treatment viability using MTT assay (Figure 2).

Compounds **9**–**16** significantly (*p* < 0.05) reduced the A549 viability after 24 h treatment in comparison to untreated control (UC). The A549 treatment with compounds at 100 µM resulted in remaining post-treatment viability ranging from 0 to 83% and the activity of the compounds was highly structure dependent. A549 cells treated with 100 µM of doxorubicin (DOX) retained 30.8% of viability, suggesting that the most potent naphthoquinone derivatives have similar or higher anticancer activity than DOX, which is often used a standard chemotherapy agent for NSCLC. The compounds **14** (38.7% of viability reduction) and **16** (28.6% of viability reduction) exerted similar cytotoxic activity to DOX (Figure 1). Interestingly, compound **9** bearing phenylamino substituent exhibited the most potent cytotoxic activity that was significantly greater than DOX’s (*p* < 0.05) (Figure 2). Furthermore, compound **9** showed minimal cytotoxic activity on non-cancerous Vero cells, suggesting that the cytotoxic activity is dependent on cancerous cells.

Furthermore, compounds **10**, **11**, **12** and **15** bearing 4-F-PhNH, 4-Cl-PhNH, 4-Br-PhNH and 4-C_2_H_5_O-PhNH moieties demonstrated moderate anticancer activity by reducing A549 viability by an average of 52–56% in comparison to UC. Compounds **10**, **12** and **15** exhibited low toxicity to Vero cells, while compound **11** (R: 4-Cl-PhNH) significantly reduced the Vero viability to 53%. Amongst all tested naphthoquinones, compound **13** bearing 4-CH_3_-PhNH moiety exhibited the lowest cytotoxic activity on both A549 and Vero cells (Figure 2).

These data suggest that phenylamino substitution in naphthoquinone nuclei is important for selective anticancer activity.

### 2.3. Naphthoquinone Derivatives Exert Structure- and Dose-Dependent Anticancer Activity on A549 Cells

**Figure 3:**
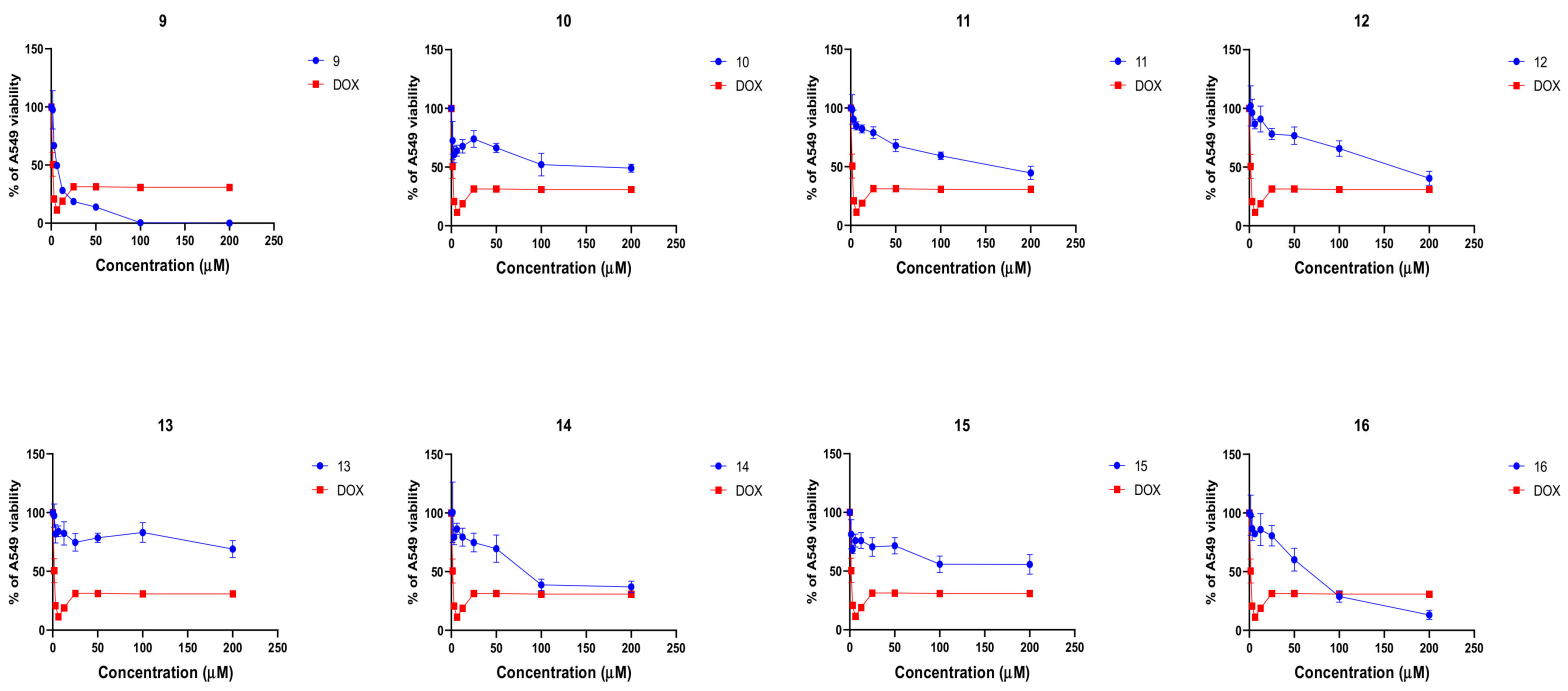
Naphthoquinone derivatives **9**–**16** are able to reduce viability in A549 non-small cell lung carcinoma cells in vitro in a concentration- and structure-dependent manner. The A549 cells were treated with compounds **9**–**16** and doxorubicin (DOX) for 24 h at concentrations of 0–200 µM and A549 viability was evaluated by using MTT assay. The data from viability is expressed as percent of viability calculated from an untreated control (UC). Data shown are mean ± SD values from three separate experiments for each group.

After confirming that the naphthoquinone derivatives exert selective anticancer activity on A549 cells, we further evaluated the dose–response kinetics of each compound. We treated the A549 cells with increasing concentrations (0–200 µM) of each compound or DOX for 24 h and then we evaluated post-treatment viability as described before. Compounds **9** (IC_50_ = 5.8 µM) and **16** (IC_50_ = 20.6 µM) exhibited the most promising concentration-dependent anticancer activity (Figure 3).

Compounds **10**–**15** demonstrated moderate-to-low micromolar cytotoxic activity (IC_50_ 38.5–146.2 µM). These results show that the presence of Ph substituent in the 3-position of the naphthoquinone ring is important for biological antiproliferative activity and that additional substitutions greatly decrease anticancer activity.

### 2.4. Naphthoquinone Derivatives **9** and **16** Are Potent Agents In Vitro Reducing the A549 Spheroid Viability

After observing the promising anticancer activity of naphthoquinone derivatives in 2D cell culture models, we decided to further evaluate whether the anticancer activity would be retained in a tumor spheroid 3D model with limited drug permeability kinetics.

**Figure 4:**
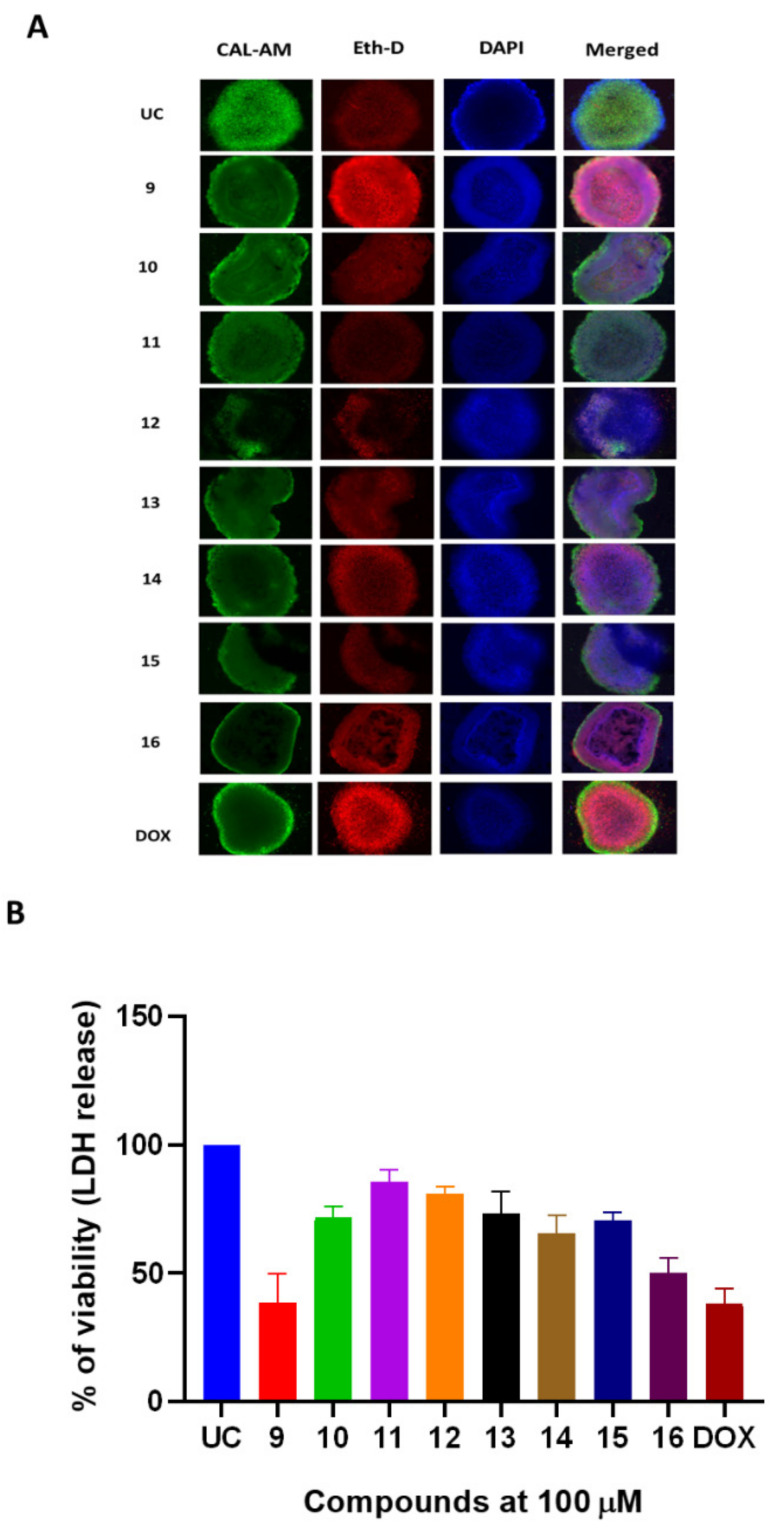
Naphthoquinone derivatives exert in vitro anticancer activity in 3D A549 NSCLC spheroid model. The A549 spheroids were treated with compounds **9**–**16** or doxorubicin (DOX) at a fixed 100 µM concentration for 24 h. (**A**) panel demonstrates the live/dead staining of A549 spheroids and the accumulation of calcein-AM (CAL-AM) by live cells as well as the accumulation of ethidium homodimer (Eth-D) by dead cells. (**B**) panel demonstrates treatment-induced cytotoxicity in A549 spheroids. Cytotoxicity was measured by quantifying LDH release. Data shown are mean ± SD values from 3 separate experiments.

The 96-h-old A549 spheroids were treated with compounds **9**–**16** or DOX for 24 h and were assayed for the cytotoxicity (Figure 4). DOX reduced 40% of viable populations at the concentration of 100 µM (Figure 4B). Doxorubicin-induced cell death was observed as the decreased calcein-AM (CAL-AM) accumulation and increased accumulation of ethidium homodimer (EthD) in spheroids as well as release of LDH (Figure 4A). Compound **16** demonstrated promising anticancer activity in spheroid model and was able to induce the LDH release similar to the DOX (Figure 4A,B). The in vitro treatment with compound **16** did not show profound effect on the spheroid boundaries. Treatment with compound **9** resulted in a similar reduction of A549 spheroid viability as evidenced by EdhD accumulation and partial loss of spheroid boundary morphology (Figure 4A,B). Treatment with DOX had minimal effect on spheroid boundary formation (Figure 4A,B).

Furthermore, naphthoquinone derivatives exhibited similar cytotoxic activity in the spheroid model, suggesting that the structures and substitutions prevalent in compound **9** and **16** are important for their biological effect.

### 2.5. Naphthoquinone Derivatives Suppress A549 Cell Migration in Wound Healing Assay

After demonstrating that compounds **9**–**16** are able to reduce A549 cell viability both in 2D and 3D tissue culture models, we further evaluated weather the compounds **9**–**16** are able to suppress metastatic A549 cell migration. We used the wound healing assay to evaluate the effect of compounds **9**–**16** on A549 migration and healing.

**Figure 5:**
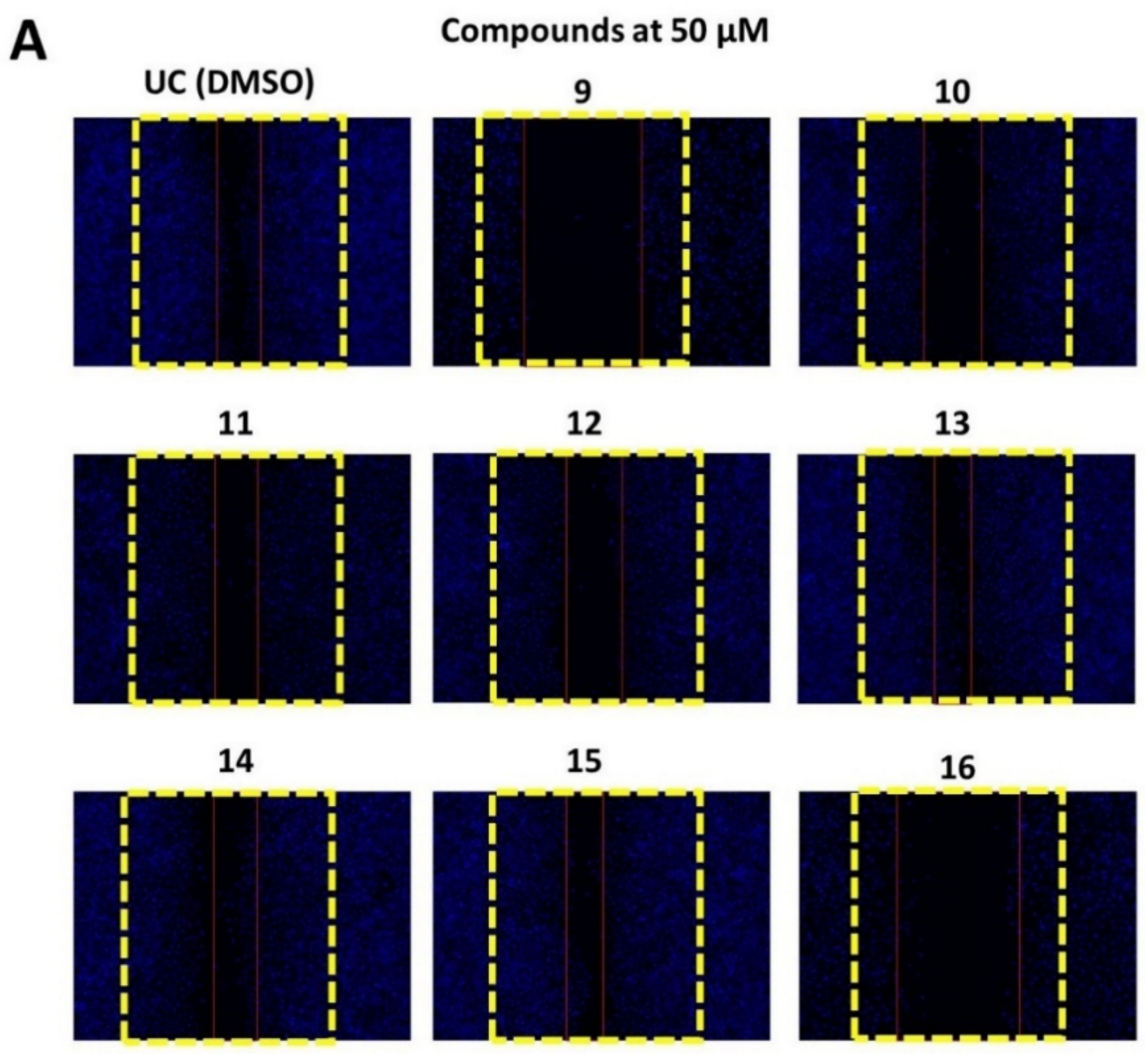

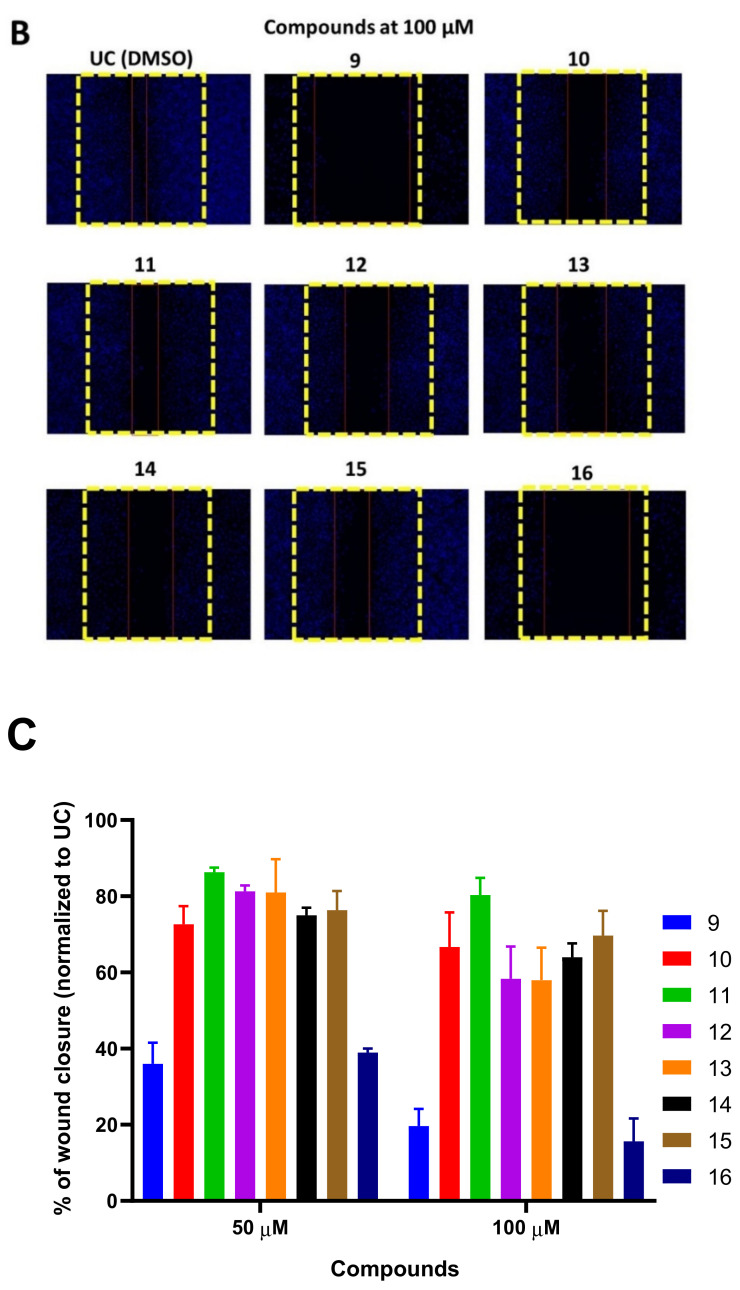
Naphthoquinone derivatives **9**–**16** are able to suppress A549 cell migration in a wound healing assay. The wound in the A549 monolayer was induced as described elsewhere and then the cells were treated with 50 (panel **A**) and 100 µM (panel **B**) of compounds **9**–**16** or media with DMSO (0.5%), which served as untreated control (UC), for 24 h. After the treatment, the media was removed, and washed cells were further incubated in compound-free media for 48 h. DAPI staining was used to visualize the cell migration. The yellow lines indicate the initial wound area, while red lines indicate the cell migration area after 48 h of incubation. The A549 migration was quantified by measuring the percentage of wound closure and normalizing it to the DMSO treated control (panel **C**). Data shown are mean ± SD values from three separate experiments. Naphthoquinone derivatives are able to block A549 colony formation.

A scratch was induced on A549 monolayer and cells were treated with 50 and 100 µM of each compound for 24 h or media containing DMSO that served as a control. After the treatment, cells were further incubated in compound-free media for an additional 48 h and cell migration was visualized by DAPI staining (Figure 5).

All tested compounds were able to suppress A549 migration better than UC (Figure 5A–C). Compounds **9** and **16** demonstrated the most notable concentration-dependent effect on A549 migration (Figure 5C). Compound **9** resulted in 36% wound healing after incubation with 50 µM of compound, while 100 µM further reduced wound closure to 19.6% in comparison to DMSO treatment (*p* < 0.05) (Figure 5A–C). A549 treatment with compound **16** at 50 µM reduced wound closure to 39% in comparison to compound free control (*p* < 0.05), while 100 µM treatment resulted in a 15.6% reduction in wound closure.

On the other hand, compounds **11**–**15** failed to significantly reduce wound closure after the treatment with 50 µM suggesting that the substitutions present in compounds **9** and **16** are critical for the anti-metastatic activity of naphthoquinone nuclei.

**Figure 6:**
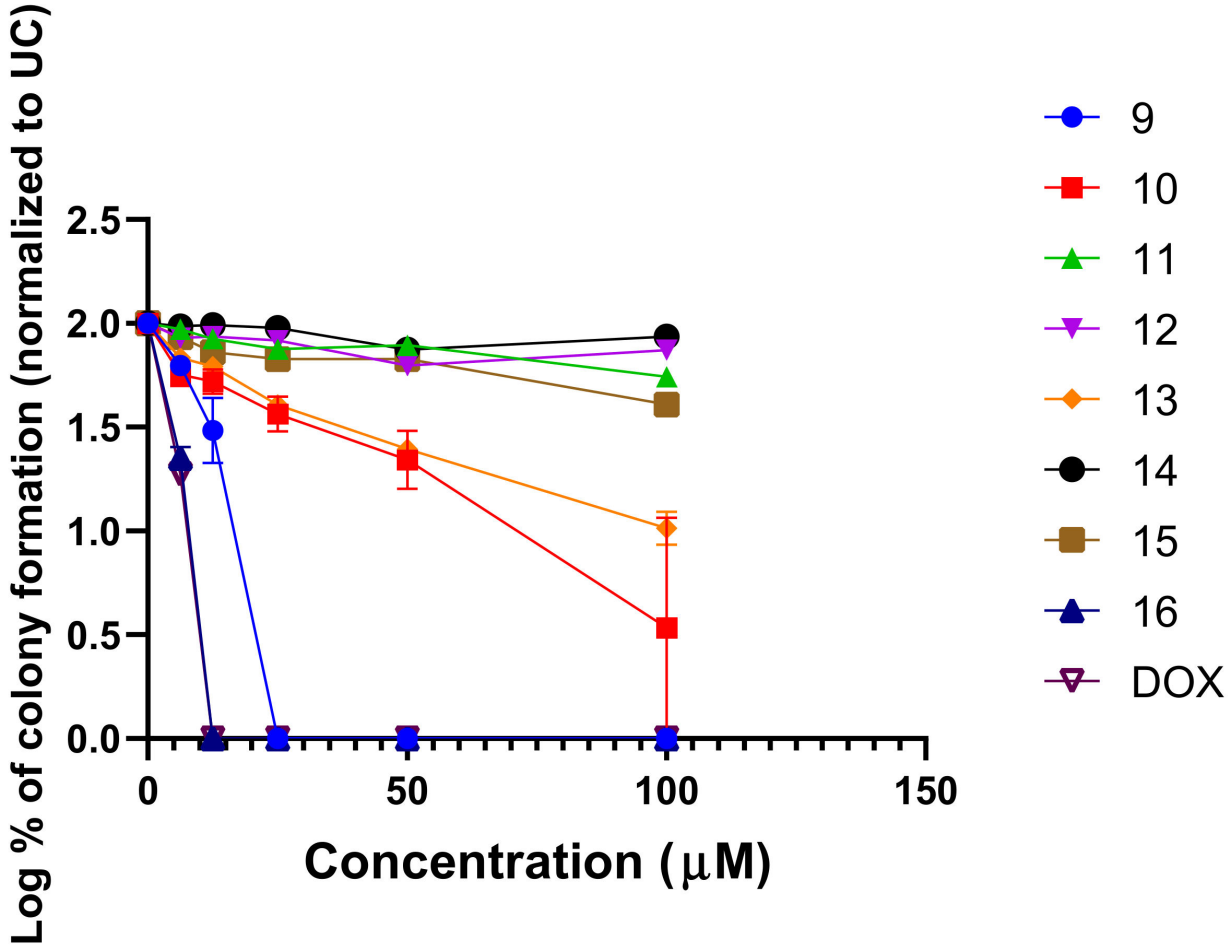
The effect of compounds **9**–**16** and doxorubicin on A549 colony formation. The cells were exposed to the test compounds or doxorubicin (DOX) for 24 h and after the treatment, colony formation was evaluated. Data shown are mean ± SD values from three separate experiments for each group.

To further characterize the cytotoxic properties of **9**–**16**, we performed the cytotoxicity assay based on A549 colony formation (Figure 6). We exposed the A549 cells to increasing concentrations of the compounds (0–100 µM) for 24 h and evaluated the effects of naphthoquinone derivatives on A549 colony formation.

Compounds demonstrated an effect on the inhibition of A549 colony formation, suggesting that naphthoquinone derivates **9**–**16** exert cytotoxic activity in the A549 NSCLC model (Figure 6). Compounds **9**, **10**, **13** and **16** exhibited the most potent inhibitory activity on A549 colony formation, suggesting possible cytotoxic and non-cytostatic activity of these compounds. Compounds **9** and **16** demonstrated the highest concentration-dependent activity on A549 colony formation inhibition (Figure 6). Compound **9** bearing PhNH substituent was able to fully suppress A549 colony formation at a concentration of 25 µM. Interestingly, compound **16** bearing 4-HO-PhNH substituent noticeably demonstrated more potent activity and was able to fully suppress A549 colony formation at a concentration of 12.5 µM. The cytotoxic activity of both compounds was comparable to that of doxorubicin.

These data further support the promising anticancer activity of naphthoquinone derivatives and demonstrate that the Ph and 4-HO-PhNH substituents are important pharmacophores mediating the cytotoxic activity.

### 2.6. Naphthoquinone Derivatives **9** and **16** Are Able to Induce Mitochondrial Injury and ROS Formation

Previous studies have demonstrated that naphthoquinone derivatives are able to induce ROS formation in various cell lines [32]. We postulate that due to high structural resemblance to ubiquinone, the cytotoxic activity of compounds **9**–**16** could potentially be mediated through the inducement of mitochondrial damage.

**Figure 7:**
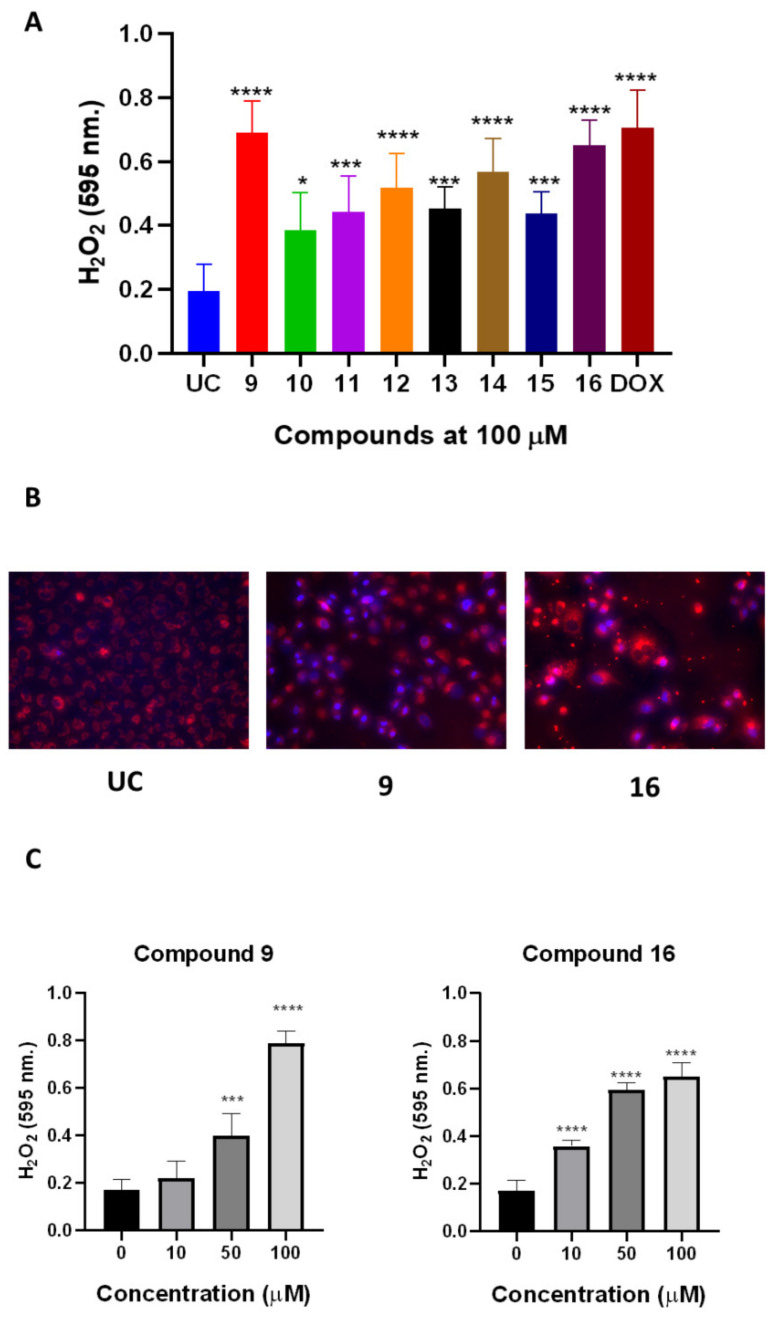
Naphthoquinone derivatives **9**–**16** are able to induce ROS production in A549 cells. The A549 spheroids were treated with compounds **9**–**16** or doxorubicin (DOX) at a fixed 100 µM concentration for 24 h and then the hydrogen peroxide was quantified using commercial assays (panel **A**,**C**). The mitochondrial injury phenotype was evaluated microscopically after staining the cells treated with compounds **9** and **19** with MitoTracker red (panel **B**). Data shown are mean ± SD values from three separate experiments for each group. The significance of the data was determined using a one-way ANOVA test. * *p* < 0.05, *** *p* < 0.0002, **** *p* < 0.0001.

We first evaluated weather treatment with compounds **9**–**16** is able to induce the production of hydrogen peroxide in A549 cells (Figure 7A). The 24 h treatment with compounds resulted in significant production of hydrogen peroxide in comparison to the untreated control (UC) (Figure 7A). A549 treatment with compounds **9** and **16** resulted in the highest (*p* < 0.05) hydrogen peroxide production in comparison to other naphthoquinone derivatives. The treatment-induced hydrogen peroxide production was similar to doxorubicin, demonstrating the similar potency in ROS post-treatment ROS induction (Figure 7A).

After showing that the compounds **9** and **16** are able to induce ROS formation, we further evaluated whether treatment results in the alteration of mitochondrial phenotype. A549 incubation with compounds **9** and **16** resulted in the mitochondrial injury phenotype, which is noticeable with staining using mitochondria-specific probes (Figure 7B). Compound **9** resulted in moderate loss of mitochondrial membrane integrity in comparison to UC, while compound **16** resulted in complete mitochondrial damage noticeable after 24 h of treatment at 100 µM (Figure 7B).

We then decided to explore the dose response of compounds **9** and **16** on ROS production by A549 cells. We treated the cells with increasing concentrations (10–100 µM) of compounds and then measured ROS production of a form of hydrogen peroxide (Figure 7C). Both compounds **9** and **16** were able to induce ROS formation in a concentration-dependent manner (Figure 7C). Notably, treatment with compound **9** resulted in greater production of hydrogen peroxide than compound **16**, suggesting that 4-HO-PhNH moiety is important for treatment-mediated ROS formation. Interestingly, despite the greater ROS production induced by compound **9**, more evident phenotypical changes in A549 mitochondria were noticeable after treatment with compound **16** (Figure 7B).

Collectively, these data demonstrate that naphthoquinone derivatives **9** and **16** can induce cell death by inducing mitochondrial damage, leading to the production of ROS. Study of structure-based activity relations has demonstrated that the phenylamino substituent in compound **9** and the 4-hydroxyphenylamino substituent in compound **16** are critical for anticancer activity.

### 2.7. Compounds **9** and **16** Display Favorable Results in Silico ADME and Drug-Likeness Profiles

**Figure 8:**
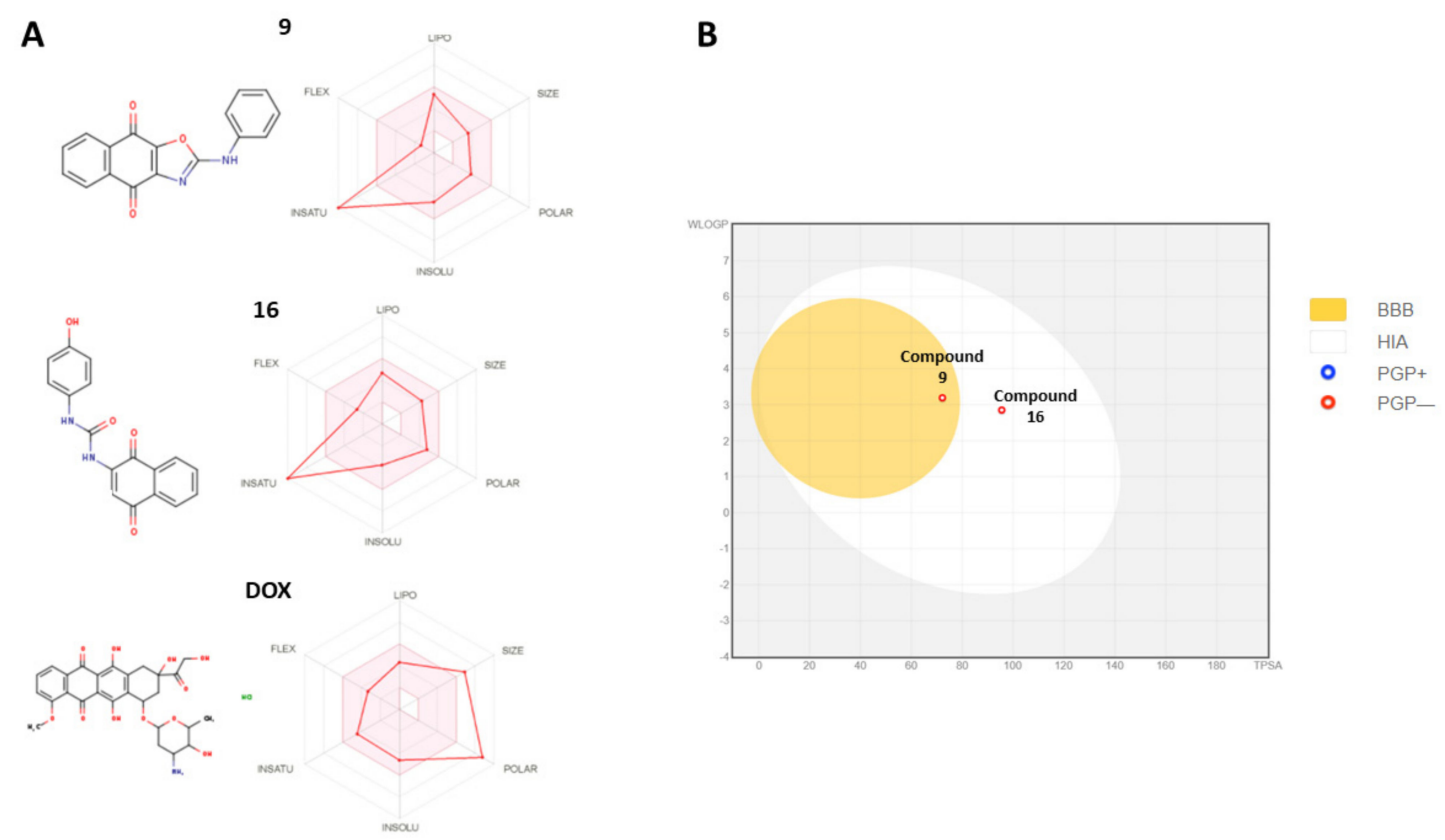
The bioavailability properties of the most potent compounds, **9** and **16,** and their comparison to doxorubicin (DOX). Panel (**A**) demonstrates the drug-likeness properties of compounds **9** and **16**. The pink area represents the optimal range for each Lipinski drug-like properties (lipophilicity: XLOGP3 between −0.7 and +5.0, size: MW between 150 and 500 g/mol, polarity: TPSA between 20 and 130 Å^2^, solubility: log *S* not higher than 6, saturation: fraction of carbons in the sp^3^ hybridization not less than 0.25, and flexibility: no more than 9 rotatable bonds). Panel (**B**) demonstrates the passive gastrointestinal absorption (HIA) and blood–brain permeability parameters. The red dots indicate that compounds **9** and **16** are not predicted to be substrates of P-gp transporter. Doxorubicin is not represented in this graph due to estimated off-limit values.

Favorable absorption, distribution, metabolism, and excretion (ADME) properties are paramount for any drug development process. Ability to use oral chemotherapeutic agents is one of the main targets in modern cancer chemotherapy. Therefore, we used an in silico prediction tool based on Lipinski drug-likeness rule to characterize the ADME properties of compounds **9** and **16** and compared them with doxorubicin (Figure 8).

Both compounds **9** and **16** demonstrated good predicted oral drug-likeness properties, making them attractive scaffolds for further hit optimization (Figure 8). The compounds had low predicted molecular weight (290.27 and 342.73 g/Mol), number of heavy atoms, ratable bonds, and H-bond donors (Table 1, Figure 8A). Doxorubicin, a standard care chemotherapy agent, demonstrated high molecular weight (579.98 g/Mol) and a higher number of heavy atoms, ratable bonds, and H-bond donors, which are critical for good gastrointestinal absorption properties (Table 1).

After demonstrating the favorable in silico predicted physicochemical properties of compounds **9** and **16**, we further predicted the pharmacokinetic distribution as well as possible excretion and metabolic interactions of compounds **9** and **16**. Both compounds **9** and **16** demonstrated high predicted gastrointestinal absorption properties (Figure 8A; Table 1). Despite high anticancer activity, doxorubicin was not predicted to be absorbable in the gastrointestinal tract (Figure 8A; Table 1).

Both compounds **9** and **16** were predicted to inhibit CYP1A2, CYP2C9, and CYP3A4 cytochrome P450 systems (Table 2). Compound **9** was predicted to inhibit CYP2C19 and CYP2D6 cytochromes, while compound **16** had no predicted inhibitory activity (Table 2).

Interestingly, only compound **9** demonstrated a good, predicted ability to cross the blood–brain barrier, suggesting a possible application of compound **9** as a brain metastasis control agent (Table 2; Figure 8B).

After demonstrating that compounds **9** and **16** bear promising anticancer activity and ADME properties, we further used in silico approaches to predict the toxicological properties of compounds **9** and **16** and their major toxicity targets. Both compounds demonstrated the variable-predicted interaction with major toxicity targets (Table 3). Compounds **9** (1250 mg/Kg) and **16** (1600 mg/Kg) demonstrated lower predicted acute toxicity (LD_50_) values in comparison to doxorubicin (LD_50_ = 205 mg/Kg), suggesting greater safety of naphthoquinone derivatives in comparison to doxorubicin. Naphthoquinone derivatives **9** and **16** showed predicted hepatotoxicity and cancerogenic activity, while doxorubicin was predicted to be inactive towards those toxicity estimations (Table 3). Doxorubicin was estimated to be immunotoxic and mutagenic, while compounds **9** and **16** were not. Compounds **9** and **16** were also estimated to interact with aryl hydrocarbon receptor (AhR). Interestingly, in silico evaluation predicted that compounds **9** and **16** would be able to interact with mitochondrial membrane potential (MMP) pathways, further supporting our experimental data collected on the mitochondrial injury phenotype.

### 2.8. Molecular Modeling and Target Prediction

**Figure 9:**
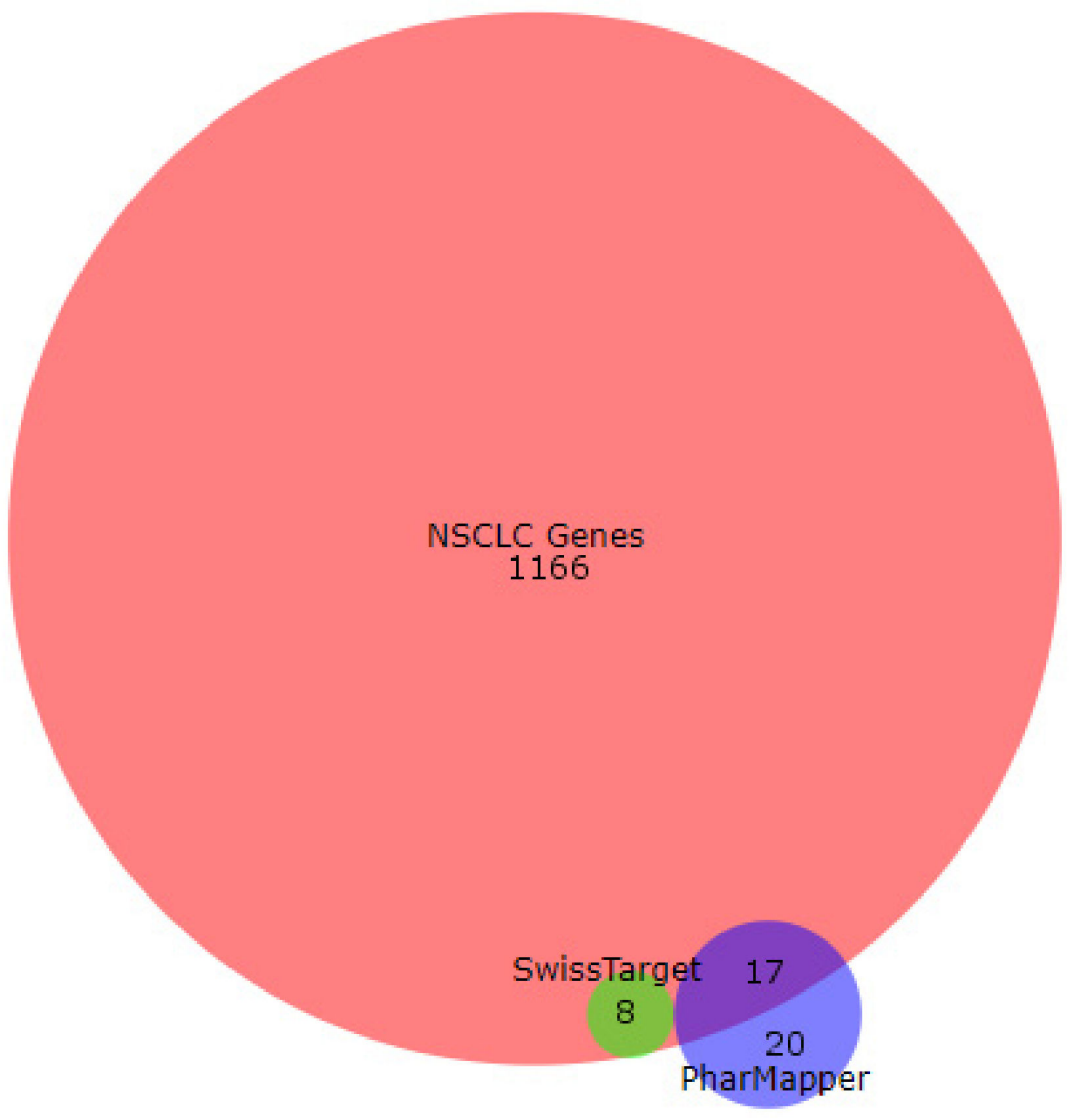
Overlapping candidate proteins between the results obtained with SwissTarget Prediction tool (in green), PharMapper tool (blue), and the non-small cancer lung cell genes obtained from GenedCards. The image was generated with BioVenn software.

Pharmacophore searching using PharMapper and SwissTargetPrediction reveals 8 and 17 as candidate proteins for the compounds **9**–**16** if they are compared with the most relevant NSCLC proteins downloaded from the GeneCards database (Figure 9).

Moreover, the most-repeated candidate proteins for all compounds are kinase proteins (66% of total overlapped proteins) (Supplementary Table S1) when using the PharMapper tool. For the SwissTargetPrediction tool, the most repeated candidate protein for compounds **9**–**16** is prostaglandin-endoperoxide synthase 2, commonly named COX-2. It is of note that this protein is also present in the GeneCard database list of proteins for this type of cell (Supplementary Table S1).

The docking results are presented in Table 4. For the case of kinases (such as ERK2, MEK1, and TPK-JAK), most of the naphthoquinones bind more strongly to MEK1 with ΔGbin values ranging from −9.4 to −8.7 kcal/mol (average -8,98 Kcal/mol). For the case of oxidoreductases (such as DFHR, TXNRD1, COX-2, NOX4, and CYP26A1), most of the naphthoquinones bind more strongly to COX-2 with ΔGbin values ranging from −9.6 to −8.7 kcal/mol (average −9.19 Kcal/mol). However, it should be noted that an optimal ΔGbin value of −9.7 Kcal/mol was found for the interaction of naphthoquinone 11 with DHFR.

**Figure 10:**
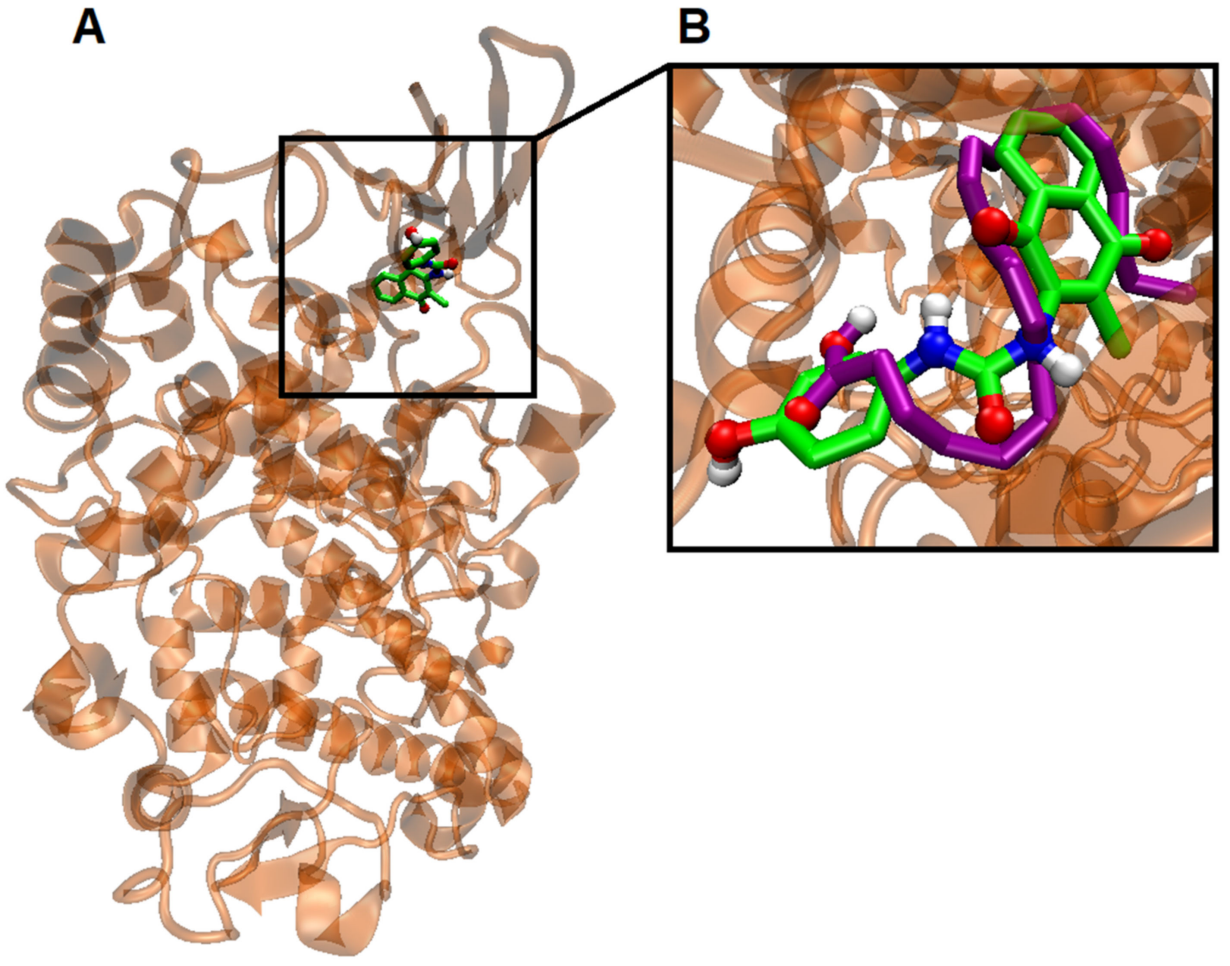
(**A**) Visualization of the potential binding site of naphthoquinone derivative **9** into COX-2, (**B**) Overlapping of the docking poses naphthoquinone **9** (green) and arachidonic acid (purple).

Furthermore, biological targets predicted by SwissTargetPrediction and PharMapper servers suggest that the COX-2 is the best NSCLC-related targets for NafoQs (Figure 10 and Supplementary Figure S1).

Taking this into consideration, docking between COX-2 protein (PDB: 3LN1) and compound **9** was performed. The results are denoted in Figure 10 and Supplementary Table S1.

**Figure 11:**
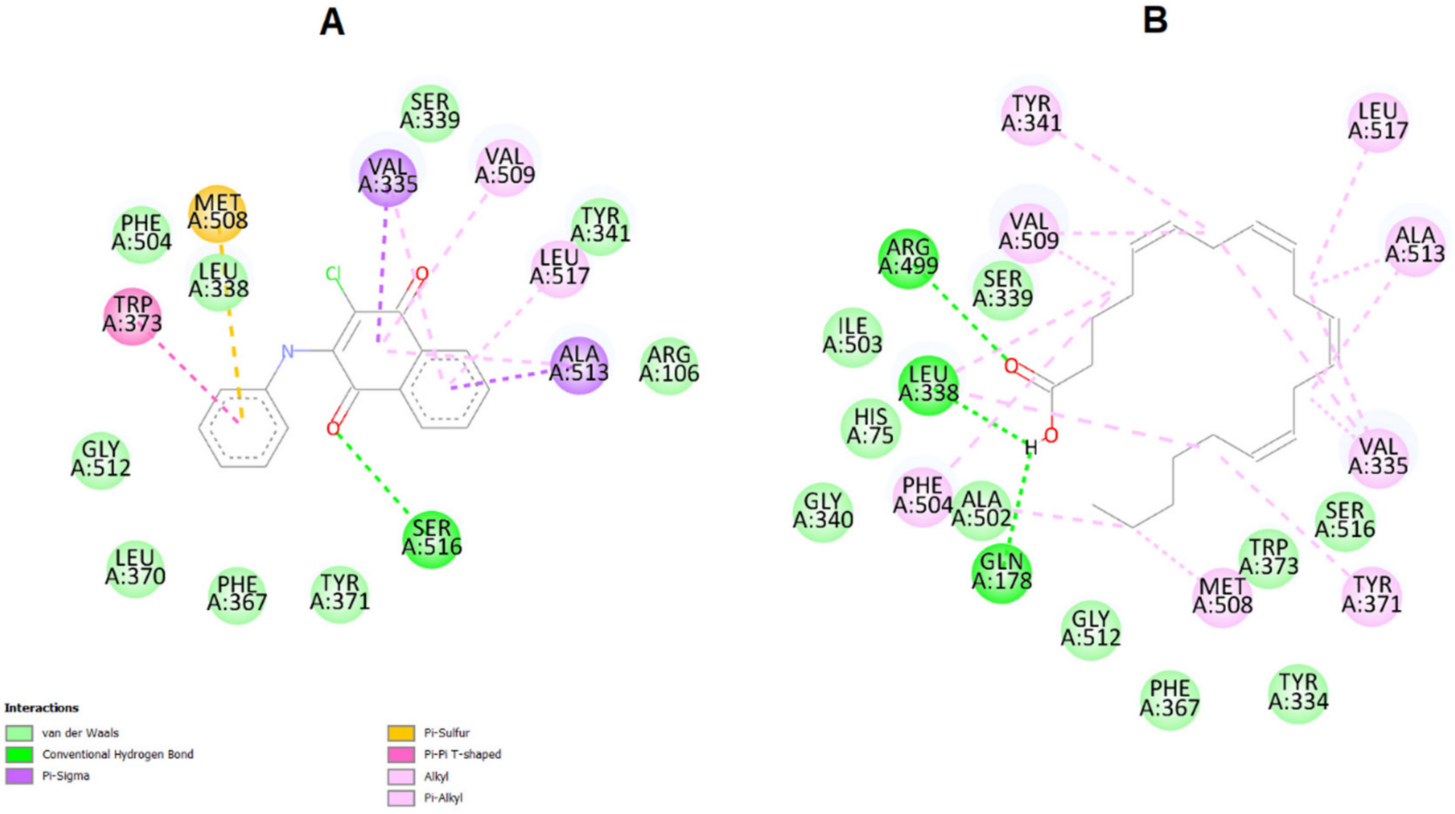
(**A**) Visualization of the interactions between COX-2 enzyme and arachidonic acid in their binding sites. Highlighted are the van der Waals interactions (pink) and hydrogen bonds (green). (**B**) Visualization of the interactions between COX-2 enzyme and compound **9**. Note that the binding site is the same as arachidonic acid. In pink: van der Waals interactions; in yellow: π-sulfur interactions; in green: hydrogen bonds (green); purple: π-σ interaction between hydrophobic amino acids (Valine 335 and Alanine 513) and the naphthoquinone moiety of compound **9**.

Interestingly, the ΔG binding energy of compound **9** is −9.4 kcal/mol, which is superior to arachidonic acid, its natural substrate (ΔGbin = −6.5 kcal/mol). Also interestingly, the potential binding sites of naphthoquinone **9** and arachidonic acid are the same (Figure 11). It should be noted that in the naphthoquinone **9**-binding region, the stabilization of the active site of COX-2 is governed mainly by van der Waals and π-σ and π-alkyl interactions, mainly established with 1,4-naphthoquinone moiety (Figure 11).

Collectively, these data suggest that the anticancer activity of the most potent compound, **9**, is mediated by the interactions with COX-2 enzyme.

## 3. Discussion

NSCLC remains one of the most common pulmonary malignancies resulting in high morbidity and mortality rates worldwide [1]. Late NSCLC diagnosis worsens the clinical prognosis and requires an aggressive treatment that is often associated with profound and systemic side effects [33,34]. In this study, we report the synthesis and in vitro anticancer activity characterization of naphthoquinone derivatives as potent compounds targeting cyclooxygenase 2 (COX-2) and inducing cell death in A549 2D and 3D NSCLC models.

Various cytotoxic and cytostatic agents alone or in combination are often used for the treatment of NSCLC. As an example, doxorubicin is anthracycline class anticancer drug targeting G (2)/M cell cycle phase and is considered a gold standard for the treatment of solid mass tumors [35,36]. Doxorubicin alone and in combination with other cytostatic compounds is often used for the treatment of localized and advanced NSCLC or prolonging the survival of end-stage NSCLC patients [37,38]. Despite high anticancer potency, doxorubicin therapy is often associated with numerous side effects, such as cardiotoxicity, pulmonary, and systemic toxicity [39,40,41]. Therefore, novel therapeutic options are necessary to overcome chemotherapy-associated side effects while still maintaining selective anticancer activity.

In this study, we identify two naphthoquinone nuclei containing hits with good anticancer activity and drug-like ADMET properties, comparable to doxorubicin in 2D and 3D A549 culture models. The most promising compounds, **9** and **16** bearing PhNH and 4-HO-PhNH moieties, demonstrated selective anticancer activity on NSCLC cells that was comparable to doxorubicin. Further substitutions in the phenylamino rig result in significant loss of anticancer activity, indicating the importance of the phenylamino group for the activity. Compounds **9** and **16** demonstrated preferably higher cytotoxic activity on cancerous cells and lower activity on non-cancerous Vero cells in comparison to doxorubicin. Interestingly, compounds **14** and **15** could potentially be a substrate for human esterase and be transformed to compounds similar to **16,** although lacking chlorine substitution. The lack of anticancer activity expressed by compounds **14** and **15** could potentially indicate that the chlorine substitution present in the structure of compound **16** is critical for the activity or that the compound itself is not activated by esterase enzymes.

Cancerous cell invasion and hematogenous and lymphatic dissemination is the first and the most crucial step in metastatic cascade leading to multiorgan involvement [42,43,44]. Transformed cells acquire the ability to leave the primary tumor location; migrate to and penetrate the surrounding tissues, lymphatic, and blood vessels; and disseminate [42,43,44]. Therefore, the pharmacological inhibition of cancerous cell migration could potentially prevent metastatic events. Naphthoquinone derivatives **9**–**16** demonstrated the ability to suppress A549 cell migration in wound healing assay in a structure- and concentration-dependent manner. The single, 24 h exposure of A549 cells with naphthoquinone derivatives resulted in altered scratch healing and cell migration profiles in comparison to untreated control. The most potent effect on A549 cell migration was observed when A549 cells were treated with compounds **9** and **16** bearing PhNH and 4-HO-PhNH moieties. These results further suggest that the PhNH and 4-HO-PhNH moieties on naphthoquinone nuclei are critical for anticancer and anti-metastatic activity.

Several studies have previously reported that naphthoquinone derivatives are able to induce ROS formation in cancer cells leading to the apoptosis and cell death [45]. The study of Wang et al. aimed to synthesize 1,4-naphthoquinone derivatives with antiproliferative properties. When cancerous cells were incubated with the most potent compounds, authors reported the increased ROS production by liver cancer cells in vitro [45]. Further studies identified 2-(4-methoxyphenylthio)-5,8-dimethoxy-1,4-naphthoquinone as a powerful ROS-inducing anticancer compound that exerts anticancer activity by interfering with MAPK and STAT3 signaling pathways [46]. The antiproliferative compound was able to decrease the phosphorylation of extracellular signal-regulated kinase (ERK) leading to the induction of apoptosis events in human gastric cancer cell lines. Moreover, Shen et al. have shown that the 2-(6-Hydroxyhexylthio)-5,8-dimethoxy-1,4-naphthoquinone is able to induce the apoptosis in human lung cancer A549 cells via same MAPK/STAT3 pathway, demonstrating the important role of naphthoquinones as a promising anticancer candidate targeting various types of tumors [47]. We have demonstrated that compounds **9**–**16** were able to induce ROS production in A549 cells in a structure-dependent manner. Treatment with compounds **9** and **16** resulted in the highest ROS production, which was comparable to doxorubicin treatment.

Ubiquinone, an important cofactor critical for oxidative phosphorylation and cellular respiration processes, shares considerable structural similarities with naphthoquinones. Several studies have demonstrated that 1,4-naphthoquinones are able to induce cell death by directly or indirectly targeting mitochondrial processes [48]. After observing a compound-mediated release of ROS, we further hypothesized that it could be potentially associated with the mitochondrial damage induced by the most potent compounds, **9** and **16**. By using fluorescence microscopy and biochemical assays coupled with mitochondria-specific probes, we demonstrated that naphthoquinone derivatives **9** and **16** are able to induce mitochondrial damage, leading to the loss of mitochondrial membrane integrity. These results further confirmed our hypothesis that the mitochondrial injury axis is involved in the cytotoxic activity of compounds **9** and **16** in a A549 model. Similar findings were observed by Majiene et al., where authors demonstrated the cytotoxic activity of various 1,4-naphthoquinone derivatives on mitochondrial function and redox potential in C6 glioblastoma cells [49]. Authors successfully showed that 1,4-naphthoquinone nuclei bearing compounds were able to induce necrotic cell death by suppressing oxidative phosphorylation in C6 glioblastoma cells leading to interference with mitochondrial respiration processes [49].

Transformed cells often have many upregulated proteins that are directly or indirectly involved in the cell survival and proliferation processes. Among those, cyclooxygenase 2 (COX-2) is highly expressed in many tumor types, and it was previously associated with cancerous cell proliferation, metastasis, and the acquisition of resistance to the anticancer drugs [50]. By using a reverse docking approach, we were able to identify the potential target for compound **16**. Further molecular modeling studies coupled with biological target prediction through web servers suggest that COX-2 is the best NSCLC-related target for the studied naphthoquinones, which could explain the mitochondrial damage induced by naphthoquinone derivatives leading to the ROS formation, as shown in Figure 7. Also, studies have demonstrated that reduced naphthoquinones catalyze the reduction of oxygen to O^2−^˙, the precursor of all biologically relevant ROS, including H_2_O_2_. Additionally, experimental evidence has shown a relationship between ROS and COX-2-derived prostanoids in cardiovascular system cells, i.e., ROS can activate COX-2 and the COX/PG (prostaglandin) synthase pathways can induce ROS production through effects on different ROS-generating enzymes, such as NADPH oxidase [51]. In this context, we propose that decreasing prostanoids, such as PGI2 and PGE2, with naphthoquinone-inhibited COX-2 could induce ROS production by increasing NADPH oxidase activity. Further experimental studies are needed to confirm our proposed mechanism of action.

In this study, we identified a new and promising anticancer candidate with good and selective activity against NSCLC in vitro. Profound and low micromolar activity displayed by compounds **9** and **16** makes them attractive scaffolds for further hit optimization and pre-clinical development. Further studies are needed to better understand safety, pharmacological activity, and in vivo efficacy of compounds **9** and **16** on NSCLC.

## 4. Materials and Methods

### 4.1. Chemistry

Reagents and solvents were obtained from Sigma-Aldrich (St. Louis, MO, USA) and used without further purification. The reaction course and purity of the synthesized compounds were monitored by TLC using aluminum plates precoated with Silica gel with F254 nm (Merck KGaA, Darmstadt, Germany). Melting points were determined with a B-540 melting point analyzer (Büchi Corporation, New Castle, DE, USA) and were uncorrected. NMR spectra were recorded on a Brucker Avance III (400, 101 MHz) spectrometer. Chemical shifts were reported in (δ) ppm relative to tetramethylsilane (TMS) with the residual solvent as internal reference ([D_6_]DMSO, δ = 2.50 ppm for ^1^H and δ = 39.5 ppm for ^13^C). Data were reported as follows: chemical shift, multiplicity, coupling constant (Hz), integration, and assignment. IR spectra (υ, cm^−1^) were recorded on a Perkin–Elmer Spectrum BX FT–IR spectrometer using KBr pellets. Elemental analyses (C, H, N) were conducted using the Elemental Analyzer CE-440; their results were found to be in good agreement (±0.3%) with the calculated values.

#### 4.1.1. General Procedures for the Synthesis of Compounds **9**–**16**

To a solution of corresponding *N*-substituted urea **1**–**8** (3 mmol), hot water (25 mL) and 2,3-dichloro-1,4-naphthoquinone (3 mmol) were added, and then sodium carbonate (5.6 mmol) was poured in. The reaction mixture was heated at reflux for 10 h, and then cooled down; the obtained solid was filtered off and recrystallized from 2-propanol to give compounds **9**–**16**.

#### 4.1.2. 2-Chloro-3-(phenylamino)-1,4-naphthoquinone (**9**)

Yield 0.75 g (88%), red solid powder; m.p. 211–212 °C (Lit. [19]: m. p. 210 °C).

^1^H NMR (400 MHz, DMSO-*d*_6_, δ, m. d.): 7.13 (d, 3H, *J* = 7.6 Hz, H_Ar_); 7.31 (t, 2H, *J* = 7.6 Hz, H_Ar_); 7.83 (dt, 2H, *J* = 24.8; 7.5 Hz, H_Ar_); 8.03 (dd, 2H, *J* = 7.4; 2.1 Hz, H_Ar_); 9.30 (s, 1H, NH).

^13^C NMR (101 MHz, DMSO-*d*_6_, δ, m. d.): 114.23; 123.95; 124.37; 126.07; 126.51; 127.05; 127.90; 130.24; 130.94; 131.94; 133.17; 134.63; 134.77; 138.81; 143.11 (C_Ar_); 176.66; 180.09 (2x C=O).

IR (KBr) ν_max_: 3235 (NH), 1675, 1667 (CO) cm^−1^. Calcd. for C_16_H_10_ClNO_2_, %: C 67.74; H 3.55; N 4.94. Found, %: C 67.69; H 3.50; N 4.93.

#### 4.1.3. 2-Chloro-3-(4-fluorophenylamino)-1,4-naphthoquinone (**10**)

Yield 0.90 g (99%), red solid powder; m.p. 234–235 °C (Lit. [1]: m. p. 232).

^1^H NMR (400 MHz, DMSO-*d*_6_, δ, m. d.): 6.61–6.78 (m, 4H, H_Ar_); 7.38 (dt, 2H, *J* = 25.0; 7.4 Hz, H_Ar_); 7.58 (dd, 2H, *J* = 6.6; 2.3 Hz, H_Ar_); 8.84 (s, 1H, NH).

^13^C NMR (101 MHz, DMSO-*d*_6_, δ, m. d.): 113.70; 114.50; 114.73; 126.04; 126.06; 126.12; 126.52; 130.23; 131.95; 133.18; 134.80; 135.21; 135.24; 143.39; 158.00; 160.40 (C_Ar_); 176.68; 180.03 (2x C=O).

IR (KBr) ν_max_: 3254 (NH), 1674, 1665 (CO) cm^−1^. Calcd. for C_16_H_9_ClFNO_2_, %: C 63.70; H 3.01; N 4.64. Found, %: C 63.61; H 3.00; N 4.60.

#### 4.1.4. 2-Chloro-3-(4-chlorophenylamino)-1,4-naphthoquinone (**11**)

Yield 0.93 g (98%), red solid powder; m.p. 261–262 °C (Lit. [1]: m. p. 262 °C).

^1^H NMR (400 MHz, DMSO-*d*_6_, δ, m. d.): 7.10 (d, 2H, *J* = 7.9 Hz, H_Ar_); 7.25–7.39 (m, 2H, H_Ar_);

7.82 (dt, 2H, *J* = 26.7; 7.6 Hz, H_Ar_); 8.02 (t, 2H, *J* = 6.7 Hz, H_Ar_); 9.37 (s, 1H, NH).

^13^C NMR (101 MHz, DMSO-*d*_6_, δ, m. d.): 114.55; 124.99; 126.02; 126.47; 127.79; 127.87; 130.40; 131.92; 134.69; 138.45; 143.56 (C_Ar_); 176.67; 180.01 (2x C=O).

IR (KBr) ν_max_: 3233 (NH), 1675, 1666 (CO) cm^−1^. Calcd. for C_16_H_9_Cl_2_NO_2_, %: C 60.40; H 2.85; N 4.40. Found, %: C 60.37; H 2.81; N 4.35.

#### 4.1.5. 2-Chloro-3-(4-bromophenylamino)-1,4-naphthoquinone (**12**)

Yield 0.97 g (89%), red solid powder; m.p. 270–271 °C (Lit. [1]: m. p. 268 °C).

^1^H NMR (400 MHz, DMSO-*d*_6_, δ, m. d.): 6.62 (d, 2H, *J* = 7.7 Hz, H_Ar_); 7.02 (d, 2H, *J* = 7.7 Hz, H_Ar_); 7.37 (dt, 2H, *J* = 26.7; 7.5 Hz, H_Ar_); 7.57 (d, 2H, *J* = 7.6 Hz, H_Ar_); 8.87 (s, 1H, NH).

^13^C NMR (101 MHz, DMSO-*d*_6_, δ, m. d.): 113.55; 124.81; 126.00; 126.35; 127.54; 127.71; 130.41; 131.90; 133.69; 134.57; 138.31; 143.13 (C_Ar_); 176.51; 180.11 (2x C=O).

IR (KBr) ν_max_: 3242 (NH), 1676, 1667 (CO) cm^−1^. Calcd. for C_16_H_9_BrClNO_2_, %: C 53.00; H 2.50; N 3.86. Found, %: C 52.98; H 2.47; N 3.81.

#### 4.1.6. 2-Chloro-3-(4-methylphenylamino)-1,4-naphthoquinone (**13**)

Yield 0.86 g (97%), red solid powder; m.p. 201–202 °C (Lit. [21]: 202 °C).

^1^H NMR (400 MHz, DMSO-*d*_6_, δ, m. d.): 2.29 (s, 3H, CH_3_); 7.00 (d, 2H, *J* = 8.0 Hz, H_Ar_); 7.11 (d, 2H, *J* = 8.0 Hz, H_Ar_); 7.78 (t, 1H, *J* = 7.5 Hz, H_Ar_); 7.85 (t, 1H, *J* = 7.5 Hz, H_Ar_); 8.01 (dd, 2H, *J* = 7.7; 2.8 Hz, H_Ar_); 9.25 (s, 1H, NH).

^13^C NMR (101 MHz, DMSO-*d*_6_, δ, m. d.): 20.55 (CH_3_); 99.52; 113.02; 123.92; 125.98; 126.45; 128.39; 130.27; 132.23; 132.91; 133.44; 134.73; 143.52 (C_Ar_);176.13; 180.25 (2x C=O).

IR (KBr) ν_max_: 3224 (NH), 1676, 1670 (CO) cm^−1^. Calcd. for C_17_H_12_ClNO_2_, %: C 68.58; H 4.06; N 4.70. Found, %: C 68.55; H 4.03; N 4.67.

#### 4.1.7. 2-Chloro-3-(4-methoxyphenylamino)-1,4-naphthoquinone (**14**)

Yield 0.90 g (96%), violet solid powder; m.p. 223–224 °C (Lit. [1]: m. p. 222 °C).

^1^H NMR (400 MHz, DMSO-*d*_6_, δ, m. d.): 3.75 (s, 3H, OCH_3_); 6.86 (d, 2H, *J* = 8.5 Hz, H_Ar_); 7.00 (d, 2H, *J* = 8.4 Hz, H_Ar_); 7.73 (t, 1H, *J* = 7.5 Hz, H_Ar_); 7.82 (t, 1H, *J* = 7.5 Hz, H_Ar_); 7.98 (dd, 2H, *J* = 12.6; 7.6 Hz, H_Ar_); 9.20 (s, 1H, NH).

^13^C NMR (101 MHz, DMSO-*d*_6_, δ, m. d.): 55.20 (OCH_3_); 113.18; 125.18; 125.81; 126.32; 130.37; 132.41; 134.60; 156.10 (C_Ar_); 176.25; 180.49 (2x C=O).

IR (KBr) ν_max_: 3249 (NH), 1677, 1669 (CO) cm^−1^. Calcd. for C_17_H_12_ClNO_3_, %: C 65.08; H 3.86; N 4.46. Found, %: C 65.01; H 3.85; N 4.41.

#### 4.1.8. 2-Chloro-3-(4-ethoxyphenylamino)-1,4-naphthoquinone (**15**)

Yield 0.95 g (97%), violet solid powder; m.p. 195–196 °C (Lit. [20]: m. p. 197 °C).

^1^H NMR (400 MHz, DMSO-*d*_6_, δ, m. d.): 1.33 (t, 3H, *J* = 6.9 Hz, CH_3_); 4.01 (kv, 2H, *J* = 6.9 Hz, CH_2_) 6.81–6.90 (m, 2H, H_Ar_); 7.02–7.10 (m, 2H, H_Ar_); 7.82 (dt, 2H, *J* = 27.6; 7.5 Hz, H_Ar_); 8.02 (dd, 2H, *J* = 7.7; 3.7 Hz, H_Ar_); 9.21 (s, 1H, NH).

^13^C NMR (101 MHz, DMSO-*d*_6_, δ, m. d.): 14.70 (CH_3_); 63.16 (CH_2_); 112.16; 113.60; 126.01: 126.48; 130.12; 131.45; 132.10; 133.00; 134.83; 143.39; 155.94 (C_Ar_); 176.44; 180.14 (2x C=O).

IR (KBr) ν_max_: 3235 (NH), 1678, 1665 (CO) cm^−1^. Calcd. for C_18_H_14_ClNO_3_, %: C 65.96; H 4.31; N 4.27. Found, %: C 65.90; H 4.29; N 4.25.

#### 4.1.9. 2-(4-Hydroxyphenylamino)-1,4-naphthoquinone (**16**)

Yield 0.73 g (91%), dark red solid powder; m.p. 240–241 °C (Lit. [4]: m. p. 243 °C).

^1^H NMR (400 MHz, DMSO-*d*_6_, δ, m. d.): 5.87 (s, 1H, H_Ar_); 6.83 (d, 2H, *J* = 8.7 Hz, H_Ar_); 7.16 (d, 2H, *J* = 8.7 Hz, H_Ar_); 7.75 (t, 1H, *J* = 7,4 Hz, H_Ar_); 7.83 (t, 1H, *J* = 7,4 Hz, H_Ar_); 7.93 (d, 1H, *J* = 7.5 Hz, H_Ar_); 8.03 (d, 1H, *J* = 7.5 Hz, H_Ar_); 9.06 (s, 1H, NH); 9.57 (s, 1H, OH).

^13^C NMR (101 MHz, DMSO-*d*_6_, δ, m. d.): 100.75; 115.79; 125.23; 125.78; 126.01; 128.95; 130.43; 132.39; 132.82; 134.84; 147.04; 155.32 (C_Ar_); 181.70; 182.08 (2x C=O).

IR (KBr) ν_max_: 3303 (NH), 1673, 1627 (CO) cm^−1^. Calcd. for C_16_H_11_NO_3_, %: C 72.45; H 4.18; N 5.28. Found, %: C 72.43; H 4.17; N 5.27.

### 4.2. Single Crystal X-ray Diffraction Analysis

For compounds **9** and **12**, diffraction data were collected at low temperature (220.0(1) K) on an automatic four-circle diffractometer single-crystal X-ray diffractometer Rigaku, XtaLAB Synergy, Dualflex, HyPix using Cu K_α_ monochromatic radiation (λ = 1.54184 Å). The crystal structures were solved by direct methods with the ShelXT [52] structure solution program using intrinsic phasing and refined with the SHELXL refinement package [53]. All calculations were performed with the help of Olex2 software [54]. Table 5 lists the main crystal data for these compounds.

### 4.3. Computational Prediction of ADMET Properties

The absorption distribution, metabolism, and excretion (ADME) properties were predicted by using SwissADME software with default settings [55,56]. The oral toxicity and toxicity targets were predicted using ProTox-II software [57,58]. The SMILE structures were generated and used for ADME prediction. Physicochemical properties, such as molecular weight, number of aromatic heavy atoms, Csp3 fraction, number of rotatable bonds, number of hydrogen bond acceptors and donors, molar refractivity, and total polar surface (TPSA) were predicted. Pharmacological properties, such as consensus lipophilicity (Log P_o/w_), water solubility (mol/L), gastrointestinal absorption, permeability through the brain-blood barrier, interactions with P-glycoprotein 1 (Pgp) and cytochromes, and skin permeability (Log Kp) were estimated.

### 4.4. Cell Lines and Culture Conditions

The non-small cell human lung carcinoma A549 and Vero cells were obtained from American Type Culture Collection (Rockville, MD, USA). Cells were maintained in Dulbecco’s Modified Eagle Medium/Nutrient Mixture F-12 (DMEM/F-12) (Gibco, Waltham, MA, USA) supplemented with 10% fetal bovine serum (10% FBS) (Gibco, Waltham, MA, USA) and 100 U/mL penicillin and 100 μg/mL streptomycin (P/S). Cells were cultured at 37 °C in a humidified atmosphere containing 5% CO_2_. Cells were fed every 2–3 days and subculture upon reaching 70–80% confluence.

### 4.5. Cell Viability Assay

The in vitro inhibitory effects of the compounds were measured by MTT assay [59,60]. Briefly, cells were plated in 96-well plates at a density of 1 × 10^4^ cells/well. After overnight attachment at 37 °C, 5% CO_2_, cells were treated with compounds (0–200 µM) in triplicate. After 20 h treatment, the MTT reagent was added, and cells were further incubated for 4 h. The formazan was then extracted with anhydrous DMSO. The samples were measured using a microplate reader at a wavelength of 570 nm. The following formula was used to calculate the percentage of A549 viability: ([AE-AB]/[AC-AB]) × 100%. AE, AC, and AB were defined as the absorbance of experimental samples, untreated samples, and blank controls, respectively. The data were analyzed using GraphPad’s GraphPad Prism or QuickCalcs.

### 4.6. Three-Dimensional Culture Spheroid Model

A549 tumor spheroids were grown as described elsewhere with modifications [61]. A549 cells were plated at a density of 2.5 × 10^4^ cells/well on the ultra-low attachment 96-well plates. After culturing for 96 h, the spheroids were treated for 24 h with 100 µM of compounds dissolved in DMEM/F12 supplemented with 10% FBS and 0.25% DMSO. The viability of the spheroids was evaluated by LDH release assay. The spheroid response to the compounds was visualized by LIVE/DEAD assay (Thermo Fisher Scientific, Waltham, MA, USA).

### 4.7. Colony Formation Assay

A549 cells were seeded on 6-well plates at a density of 2 × 10^3^ cells/well and allowed to attach overnight. Then, the cells were treated with compounds (0–100 µM) or DMSO (0.25%) for 24 h. Doxorubicin (DOX) (0–100 µM) was used as a cytotoxicity control. After treatment, media with test compounds was aspirated, and cells were washed with PBS and then further incubated in fresh media for 10 days to facilitate the development of the colonies.

After incubation, the cells were fixed with methanol and colonies were stained with 0.5% crystal violet in 10% methanol. The surviving fraction was calculated as described elsewhere [61,62]. The experiment was performed in triplicate for each test condition.

### 4.8. Cell Migration Assay

A549 cells were plated at a density of 2.5 × 10^5^ cells/well on the 24 well plates. After 24–48 h, when the cells had reached 100% confluence, wounds were made in the monolayer with non-barrier autoclaved 200 µL tips. The compounds were added to reach 50 and 100 µM concentration and were incubated for 24 h. After incubation, the media with compounds were removed; cells were washed twice with DPBS; a fresh, compound-free media was added; and cells were incubated for an additional 48 h to facilitate wound closure. After incubation, cells were fixed with 4% paraformaldehyde (PFA), stained with 300 nM of DAPI for 1 h, and observed under fluorescent microscope. The percentage of wound healing was calculated by measuring the wound closure diameter by using Image J and normalizing the percentage of wound closure to untreated control.

### 4.9. Hydrogen Peroxide Production Assay

The production of hydrogen peroxide was measured by using Pierce Quantitative Peroxide Assay Kit (Thermo Fisher Scientific). The A549 cells were seeded in 6-well plates and treated with test compounds (100 µM) or DMSO (0.25%) for 24 h as described above. After the treatment, the cells were washed with ice-cold PBS, scarped, and suspended in 0.5 mL of ice-cold PBS. The cells were lysed by sonication and the production of hydrogen peroxide was determined as described by the manufacturer.

#### 4.9.1. Mitochondria Staining

A549 cells were plated on sterile coverslips to achieve 1 × 10^4^ cells and incubated overnight. After incubation, cells were treated with test compounds (100 µM) or DMSO for 24 h and stained using 50 nM of MitoTracker red 580 for 1 h at 37 °C. After staining, cells were washed with PBS and fixed with 4% paraformaldehyde solution for 10 min at room temperature. After fixation, cells were permeabilized with 0.1% Triton X-100 in PBS for 1 min and nuclei were stained with 300 nM of DAPI. Coverslips were then mounted using AntiFade mounting media, and images were acquired on a Zeiss LSM880 microscope (Jena, Germany).

#### 4.9.2. Pharmacophore Search of Protein Targets

We used two web servers to determine which protein was a possible target for naphthoquinone derivatives **9**–**16**: PharMapper and SwissTargetPrediction [63,64,65]. The parameters for PharMapper were set by default, adding in the Advanced Options for the step of Energy Minimization and using the MMFF94 Force Field. The target was Human Protein Targets Only v.2010, 2241. The predicted targets were filtered using a Z-score > 0.2. Another filter was also added: only selecting a minimum of 7 overlaps between the predicted targets for compounds **9**–**16**.

For the SwissTargetPrediction server, the SMILES codes of the compounds **9**–**16** were deposited, and the *Homo sapiens* species was selected. The first 10 predicted target proteins were selected based on 2D and 3D similarities of the compounds with the existing ones in this database [55,56]. Then, the same filter was used with PharMapper, but a minimum of 4 overlaps were considered.

Furthermore, NSCLC-related targets were taken from GeneCards database (https://www.genecards.org/, accessed on 1 September 2021) using the phrase “non-small cell lung cancer” as a keyword and filtered using only protein coding genes and a score > 25. A total of 1166 targets were retained. The predictive filtered targets of the SwissTargetPrediction and PharMapper servers were compared with the NSCLC-related targets to select the best candidate protein targets. The resulting overlap was generated using BioVenn (http://www.biovenn.nl/index.php, accessed on 1 September 2021). The selected proteins targets were used in further analysis.

#### 4.9.3. Ligand and Receptor Preparation

The 3D structure of each compound was built using the MarvinSketch JS online tool (https://marvinjs-demo.chemaxon.com/latest/demo.html, accessed on 1 September 2021), adding hydrogens, and generating the 3D topology using the OpenBabel tool [66]. The molecules were saved in mol2 format and loaded to the SwissParam server for parametrization and correction of the topology [67]. These structures were visually checked to correct some structural errors. The crystal structures of 10 selected proteins, including kinases and oxidoreductases, were retrieved from the Protein Data Bank and models of human NADPH oxidase and cytochrome P450 26A were retrieved from AlphaFold Protein Structure Database (Uniprot accession number Q9NPH5 and O43174, respectively). They are overexpressed in some malignancies, including breast, gastric, and lung carcinomas, as described in the literature [68,69,70,71,72].

#### 4.9.4. Docking of Ligand-Protein Interaction

We resorted to virtual screening using Autodock Vina, a target-specific scoring method useful for virtual screening (Trott & Olson, 2009). Naphthoquinone derivatives **9**–**16** were docked into a set of proteins to identify the NSCLC-related targets. Both ligands and proteins were prepared using AutoDock Tools version 1.5.6 (ADT) according to the AutoDock Vina High Throughput screening standard method (Trott & Olson. 2009). Gasteiger partial charges were assigned to the atoms of ligands. The AutoTors option was used to define the rotatable bonds in the ligands. The visual inspection of the results was performed using the Molecular Graphics Laboratory (MGL) Tools package. We selected a grid volume large enough to cover each receptor. Finally, graphical analysis was performed using Visual Molecular Dynamics (VMD), version 1.9.2 [72].

#### 4.9.5. Statistical Analysis

The data are expressed as a mean ± SD values from three separate experiments unless stated otherwise. The statistical significance was determined using a one-way ANOVA test. Data were considered significant when *p* < 0.05.

## Figures and Tables

**Table 1:** Physicochemical properties of naphthoquinone derivatives **9** and **16** and their comparison to standard chemotherapy agent doxorubicin.

Compound	MW	No of Heavy Atoms	No of Aromatic Heavy Atoms	Fraction Csp^3^	Rotatable Bonds	H-Bond Acceptors	H-Bond Donors	Molar Refractivity	TPSA
9	289.71	22	17	0	2	4	1	79.35	72.2
16	265.27	24	12	0	4	4	3	88.09	95.5
DOX	579.98	40	12	0.44	5	12	6	139.63	206.07

Abbreviations: DOX—doxorubicin, TPSA—total polar surface area.

**Table 2:** Pharmacokinetic and drug excretion properties of naphthoquinone derivatives **9** and **16** and their comparison to standard chemotherapy agent doxorubicin.

Compound	GI Absorption	BBB Permeant	P-gp Substrate	CYP1A2 Inhibitor	CYP2C19 Inhibitor	CYP2C9 Inhibitor	CYP2D6 Inhibitor	CYP3A4 Inhibitor
9	High	Yes	No	Yes	Yes	Yes	Yes	Yes
16	High	No	No	Yes	No	Yes	No	Yes
DOX	Low	No	Yes	No	No	No	No	No

Abbreviations: P-gp—P glycoprotein 1; BBB—blood–brain barrier; GI—gastrointestinal; CYP1A2—Cytochrome P450 1A2; CYP2C19—Cytochrome P450 2C19; CYP2C9—Cytochrome P450 2C9; CYP2D6—Cytochrome P450 2D6; CYP3A4—Cytochrome P450 3A4; DOX—doxorubicin.

**Table 3:** The in silico predicted toxicological properties of compounds **9** and **16** and their interaction with major toxicity targets in silico. The interactions were calculated using ProTox-II software with default parameters.

Toxicity Target	Compound 9		Compound 16		DOX	
	Result	Probability	Result	Probability	Result	Probability
Hepatotoxicity	Active	0.59	Active	0.58	Inactive	0.86
Carcinogenicity	Active	0.58	Inactive	0.51	Inactive	0.9
Immunotoxicity	Inactive	0.96	Active	0.66	Active	0.99
Mutagenicity	Inactive	0.52	Inactive	0.53	Active	0.98
Cytotoxicity	Inactive	0.72	Inactive	0.64	Active	0.94
Aryl hydrocarbon Receptor (AhR)	Active	0.58	Active	0.56	Inactive	0.92
Androgen Receptor (AR)	Inactive	0.92	Inactive	0.97	Inactive	0.99
Androgen Receptor Ligand Binding Domain (AR-LBD)	Inactive	0.98	Inactive	0.97	Inactive	0.55
Aromatase	Inactive	0.84	Inactive	0.86	Active	0.52
Estrogen Receptor Alpha (ER)	Inactive	0.88	Inactive	0.8	Inactive	0.73
Estrogen Receptor Ligand Binding Domain (ER-LBD)	Inactive	0.98	Inactive	0.97	Inactive	0.74
Peroxisome Proliferator Activated Receptor Gamma (PPAR-Gamma)	Inactive	0.97	Inactive	0.83	Inactive	0.97
Nuclear factor (erythroid-derived 2)-like 2/antioxidant responsive element (nrf2/ARE)	Inactive	0.96	Inactive	0.87	Inactive	0.98
Heat shock factor response element (HSE)	Inactive	0.96	Inactive	0.87	Inactive	0.98
Mitochondrial Membrane Potential (MMP)	Active	0.52	Active	0.6	Inactive	0.56
Phosphoprotein (Tumor Suppressor) p53	Inactive	0.8	Inactive	0.71	Active	0.52
ATPase family AAA domain-containing protein 5 (ATAD5)	Inactive	0.76	Inactive	0.91	Inactive	0.63

Overall, these data show that both compounds **9** and **16** display favorable predicted ADME properties. The low predicted toxicity of compound **16** and exceptional in vitro potency in cell culture models make it an attractive lead for further pre-clinical validation and optimization.

**Table 4:** Predicted binding free energy values (ΔGbin kcal/mol) for the docking of naphthoquinones with proteins overexpressed in cancer.

NafoQ	ERK2	MEK1	TPK-JAK	DHFR	TXNRD1	COX-2	NOX4	CYP26A1	*N avge.*
9	−8.3	−8.7	−7.9	−9.4	−8.0	−9.4	−9.1	−8.2	−8.63
10	−8.4	−9.0	−8.5	−9.6	−9.0	−9.6	−9.3	−8.5	−8.99
11	−8.5	−9.0	−8.4	−9.7	−7.9	−8.8	−8.9	−8.5	−8.71
12	−8.5	−9.2	−7.6	−7.8	−7.8	−9.0	−8.8	−8.1	−8.35
13	−8.2	−9.4	−8.4	−7.8	−7.6	−9.5	−9.1	−8.6	−8.58
14	−8.3	−8.7	−8.3	−7.9	−7.5	−8.7	−8.9	−8.3	−8.33
**15**	−8.3	−9.0	−8.6	−9.1	−7.3	−9.0	−8.8	−8.2	−8.54
16	−8.4	−8.8	−9.1	−9.1	−7.2	−9.5	−8.7	−8.1	−8.61
*P avge.*	−8.36	−8.98	−8.35	−8.80	−7.79	−9.19	−8.95	−8.31	

Proteins with their respective (PDB) entries: ERK2: extracellular signal-regulated kinase 2 (2OJG); MEK1: MAPK/ERK kinase (4AN3); TPK-JAK: tyrosine-protein kinase–Janus kinase (4EHZ); DHFR: dihydrofolate reductase (1DLS); TXNRD1: thioredoxin reductase 1 cytoplasmic (2ZZ0); COX-2: cyclooxygenase-2 (3LN1); NOX4: NADPH oxidase; CYP26A: cytochrome P450 26A1. *N avge.*: naphthoquinones average, mean of the DGbin values of each compound with all proteins shown; best three NafoQs are highlighted in pink and pale pink colors. *P avge.*: Protein average, mean of the DGbin values for the interactions of each protein with all the NIQs; the three proteins with the highest global naphthoquinones affinity means are highlighted on green and pale-green colors.

**Table 5:** Crystal data and structure refinement parameters for compounds **9** and **12**.

Parameter	Compound 9	Compound 12
Empirical formula	C_16_H_10_ClNO_2_	C_16_H_9_BrClNO_2_
Formula weight	283.72	362.59
Crystal size (mm^3^)	0.19 × 0.11 × 0.06	0.23 × 0.14 × 0.12
Crystal system	monoclinic	Orthorhombic
Space group	*P*2_1_/*c*	*Pna*2_1_
*a* (Å)	4.8166(1)	12.18779(9)
*b* (Å)	22.4574(4)	23.6416(2)
*c* (Å)	11.7402(2)	4.73623(3)
β (°)	98.081(2)	90
Unit cell volume (Å^3^)	1257.31(4)	1364.69(2)
Molecular multiplicity	4	4
Calculated density (g/cm^3^)	1.499	1.765
Absorption coefficient (mm^−1^)	2.693	5.940
*F*(000)	584	720
2θ_max_ (°)	155.0	155.0
Reflections collected	13,655	11,253
Number of independent reflections	2618 (*R*_int_ = 0.0279)	2259 (*R*_int_ = 0.0270)
Reflections with *I* > 2σ(*I*)	2548	2242
Number of refined parameters	185	195
Goodness of fit	1.052	1.043
*R*-factors (*R*1 for *I* > 2σ(*I*), and *wR*2 for all data)	0.0382, 0.1037	0.0260, 0.0710
Δρ_max_, Δρ_min_ (e Å^−3^)	0.245, −0.472	0.688, −0.301
CCDC deposition number	2,093,829	2,093,828

## Data Availability

Data is contained within the article and Supplementary Materials. The compounds and cell lines are available from the corresponding author.

## Supplementary Materials

The following are available online at https://www.mdpi.com/article/10.3390/ph15050541/s1. Figure S1: ^1^H-NMR spectrum of compound **9**; Table S1.
