# Supplementary material for: Synthesis, Biological Activity, and Molecular Modelling Studies of Naphthoquinone Derivatives as Promising Anticancer Candidates Targeting COX-2
Source: Pharmaceuticals (Basel). 2022 Apr 27;15(5):541. doi: 10.3390/ph15050541 (PMC9144205; doi:10.3390/ph15050541)
Supplement: Supplementary file 1 [file pharmaceuticals-15-00541-s001.zip › Kavaliauskas_2021_suplementary_fig.pdf]

Synthesis, Biological Activity and Molecular Modelling Studies of Naphthoquinone Derivatives as Promising Anticancer Candidates Targeting COX-2

Povilas Kavaliauskas [1,2,3,4,5,*], Felipe Stambuk Opazo [6], Waldo Acevedo [7], Ruta Petraitiene [2,4], Birutė Grybaitė [1], Kazimieras Anusevičius [1], Vytautas Mickevičius [1,4], Sergey Belyakov [8] and Vidmantas Petraitis [2,4,7]

* To whom the correspondence should be addressed: Povilas Kavaliauskas, Kaunas University of Technology, Department of Organic Chemistry. Radvilenu rd. 19, LT-50254 Kaunas, Lithuania Email: pok4001@med.cornell.edu

1 Department of Organic Chemistry, Kaunas University of Technology, Radvilenu Rd. 19, LT-50254 Kaunas, Lithuania; birute.grybaite@ktu.lt (B.G.); kazimieras.anusevicius@ktu.lt (K.A.); vytautas.mickevicius@ktu.lt (V.M.)

2 Joan and Sanford I. Weill Department of Medicine, Weill Cornell University, 1300 York Avenue, New York, NY 10065, USA; rop2016@med.cornell.edu (R.P.); vip2007@med.cornell.edu (V.P.)

3 Department of Microbiology and Immunology, University of Maryland School of Medicine, 655 W. Baltimore Street, Baltimore, MD 21201, USA

4 Institute of Infectious Diseases and Pathogenic Microbiology, Birstono Str. 38A, LT-59116 Prienai, Lithuania

5 Biological Research Center, Lithuanian University of Health Sciences, Tilzes Str. 18, LT-47181 Kaunas, Lithuania

6 Instituto de Biología, Facultad de Ciencias, Pontificia Universidad Católica de Valparaíso, Valparaíso. Av. Universidad N° 330, Curauma, Valparaiso 2373223, Chile; felipe.stambuk.o@mail.pucv.cl

7 Instituto de Química, Facultad de Ciencias, Pontificia Universidad Católica de Valparaíso, Valparaíso. Av. Universidad N° 330, Curauma, Valparaiso 2373223, Chile; waldo.acevedo@pucv.cl

8 Latvian Institute of Organic Synthesis, Laboratory of Physical Organic Chemistry, Aizkraukles 21, LV-1006 Riga, Latvia; serg@osi.lv

Content:

Supplemental Figures S1−S16, representing ^{1}H and ^{13}C NMR spectra of all the newly synthesized compounds **9−16**.

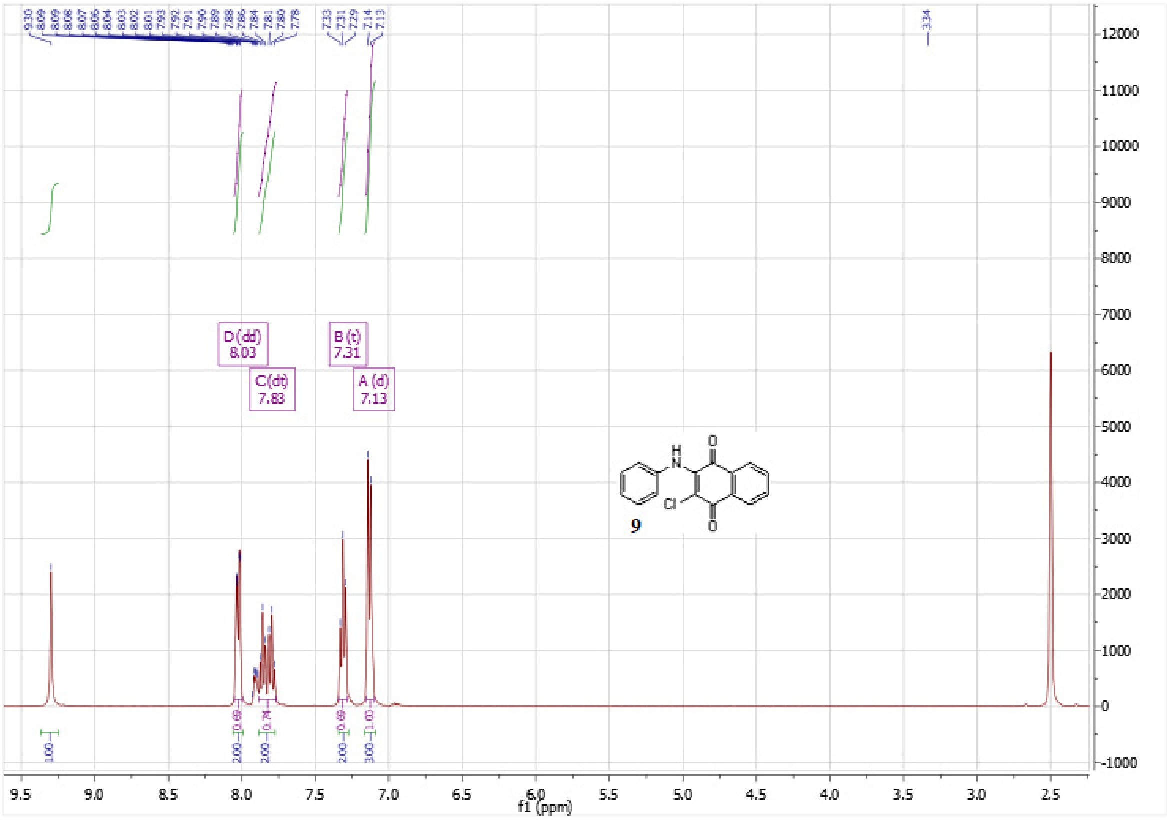

Figure S1: ^{1}H-NMR spectrum of compound **9**.

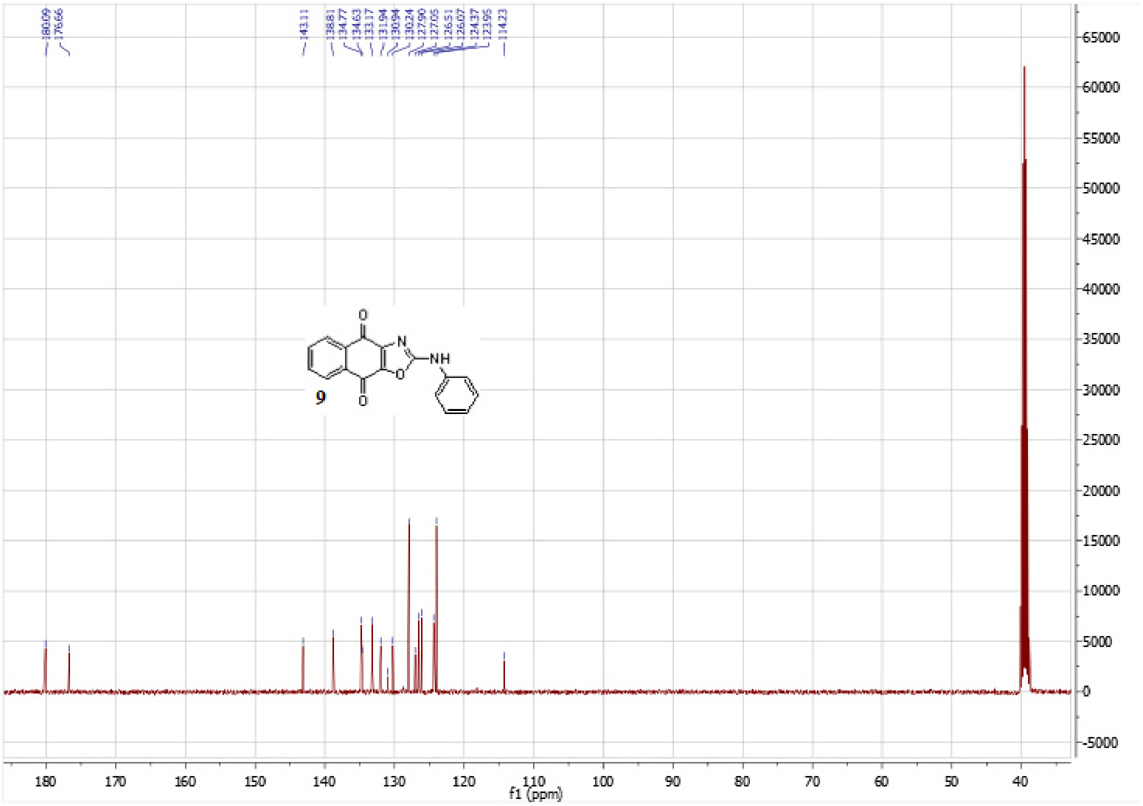

Figure S2: ^{13}C-NMR spectrum of compound **9**.

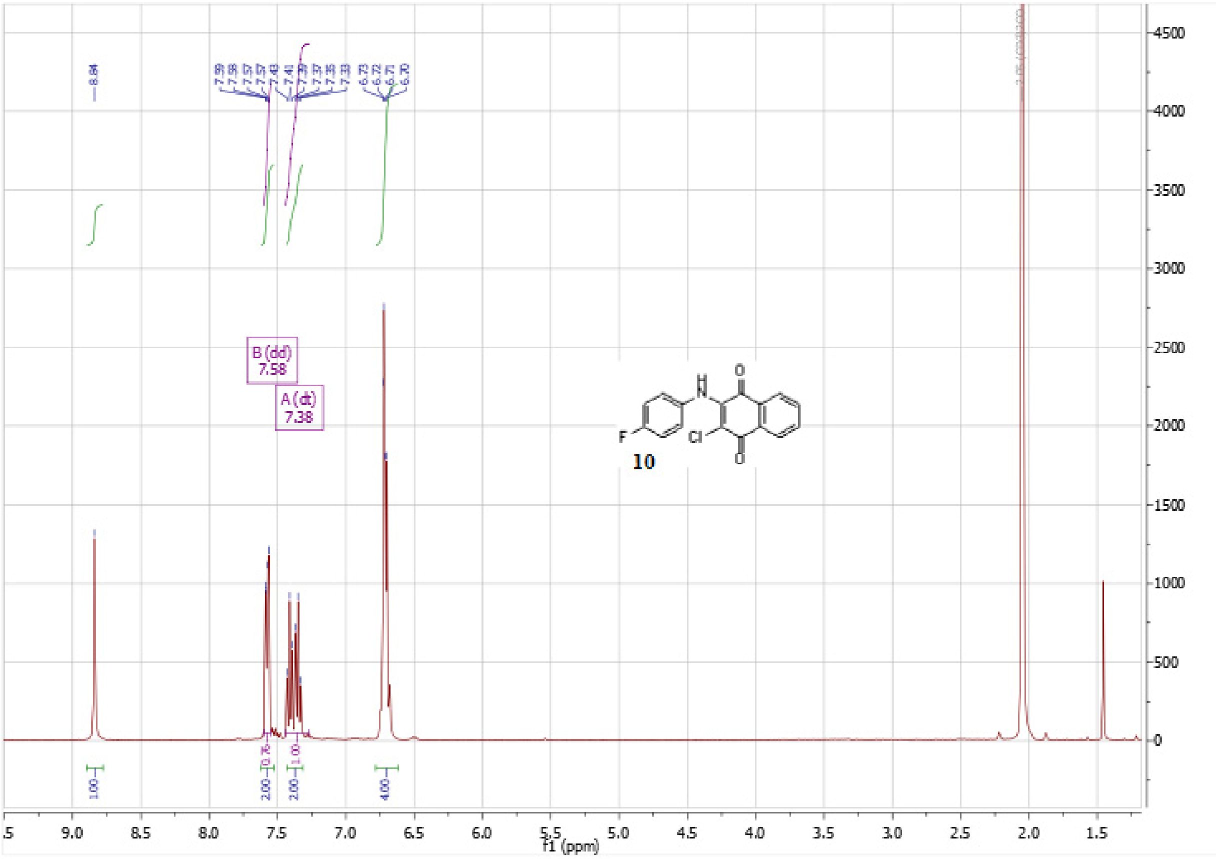

Figure S3: ^{1}H-NMR spectrum of compound **10**.

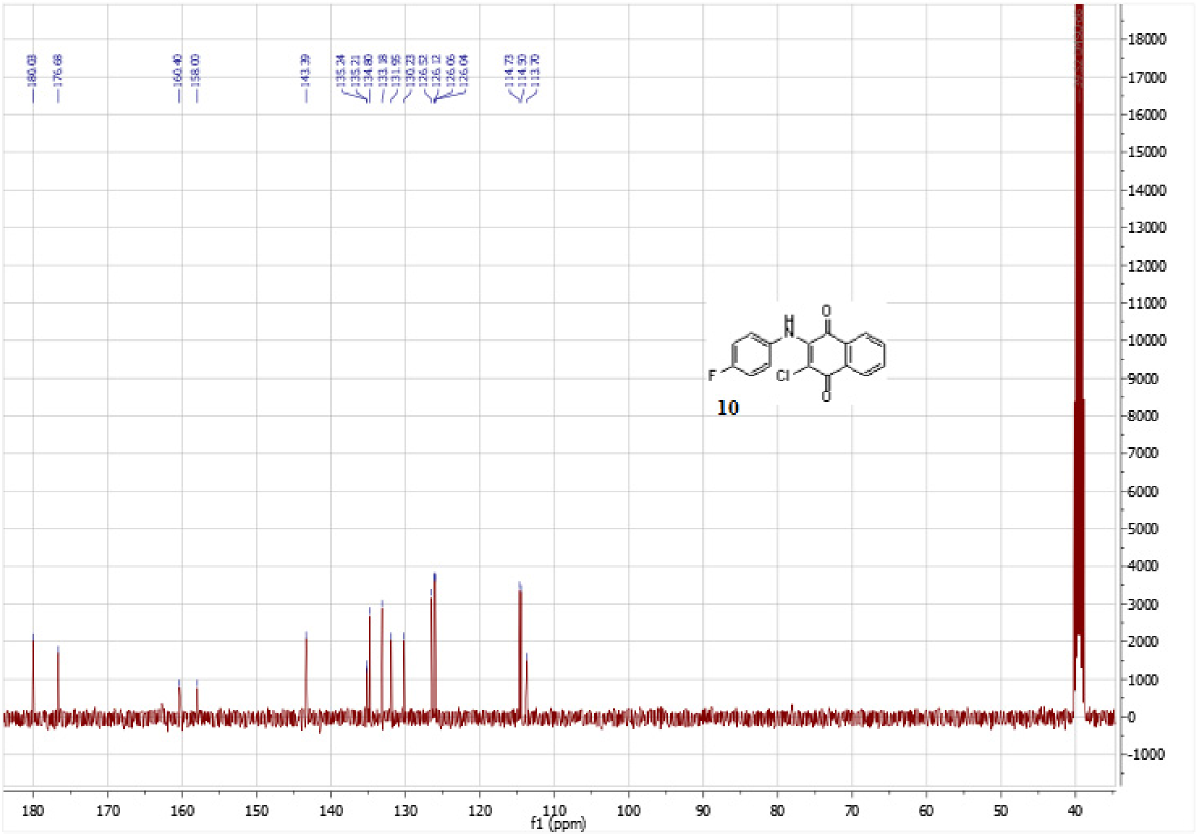

Figure S4: ^{13}C-NMR spectrum of compound **10**.

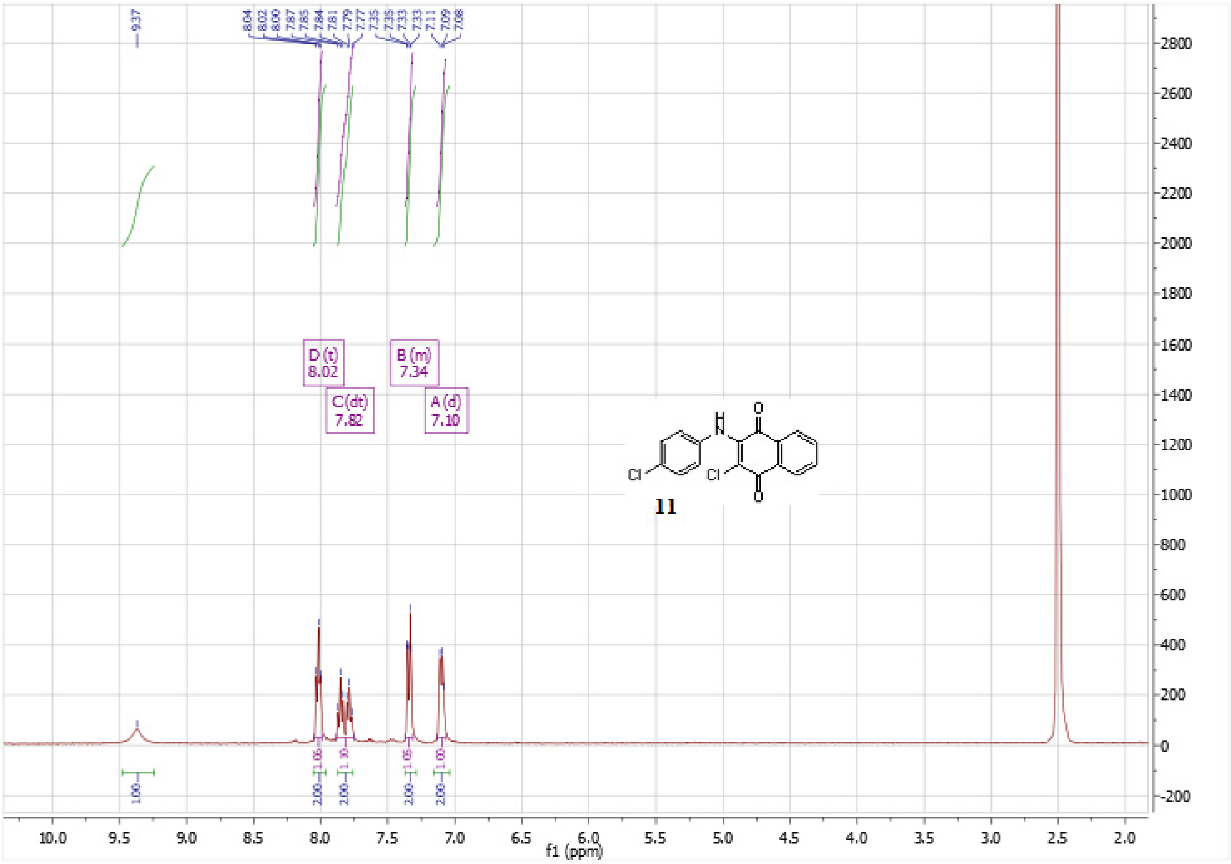

Figure S5: ^{1}H-NMR spectrum of compound **11**.

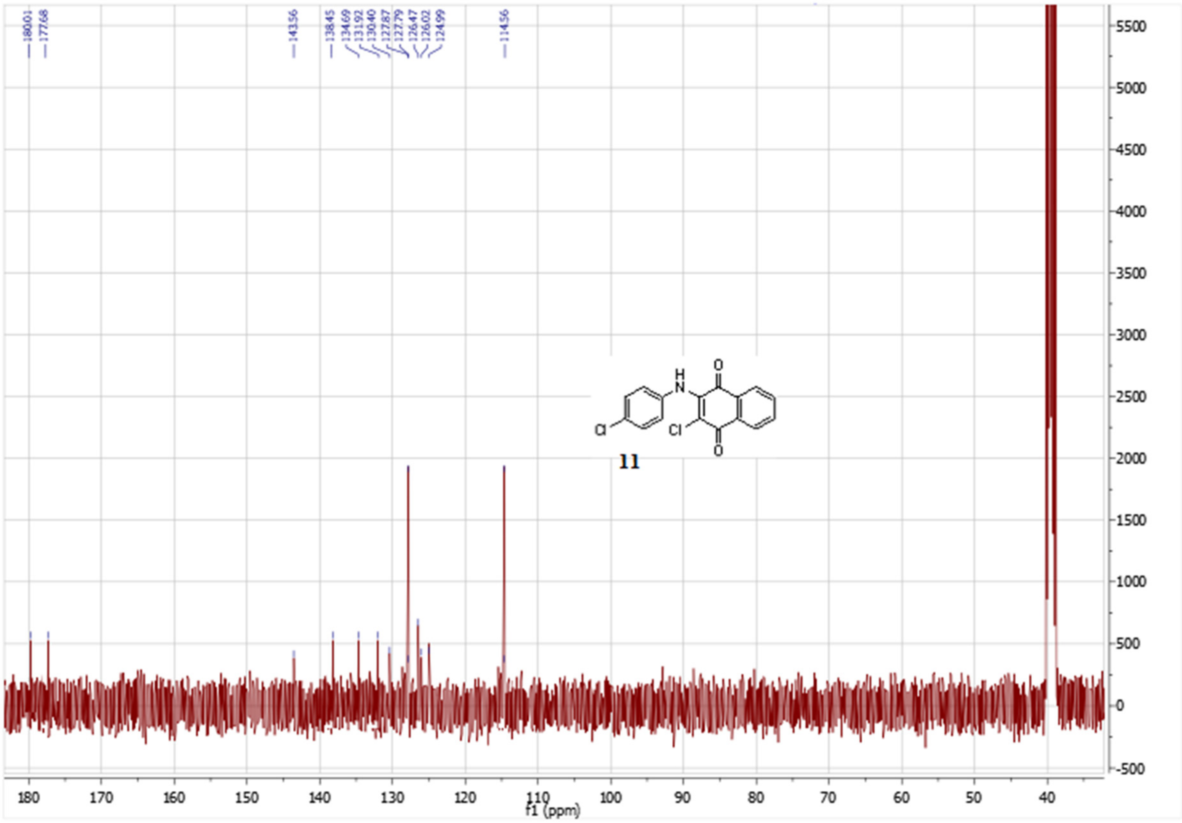

Figure S6: ^{13}C-NMR spectrum of compound **11**.

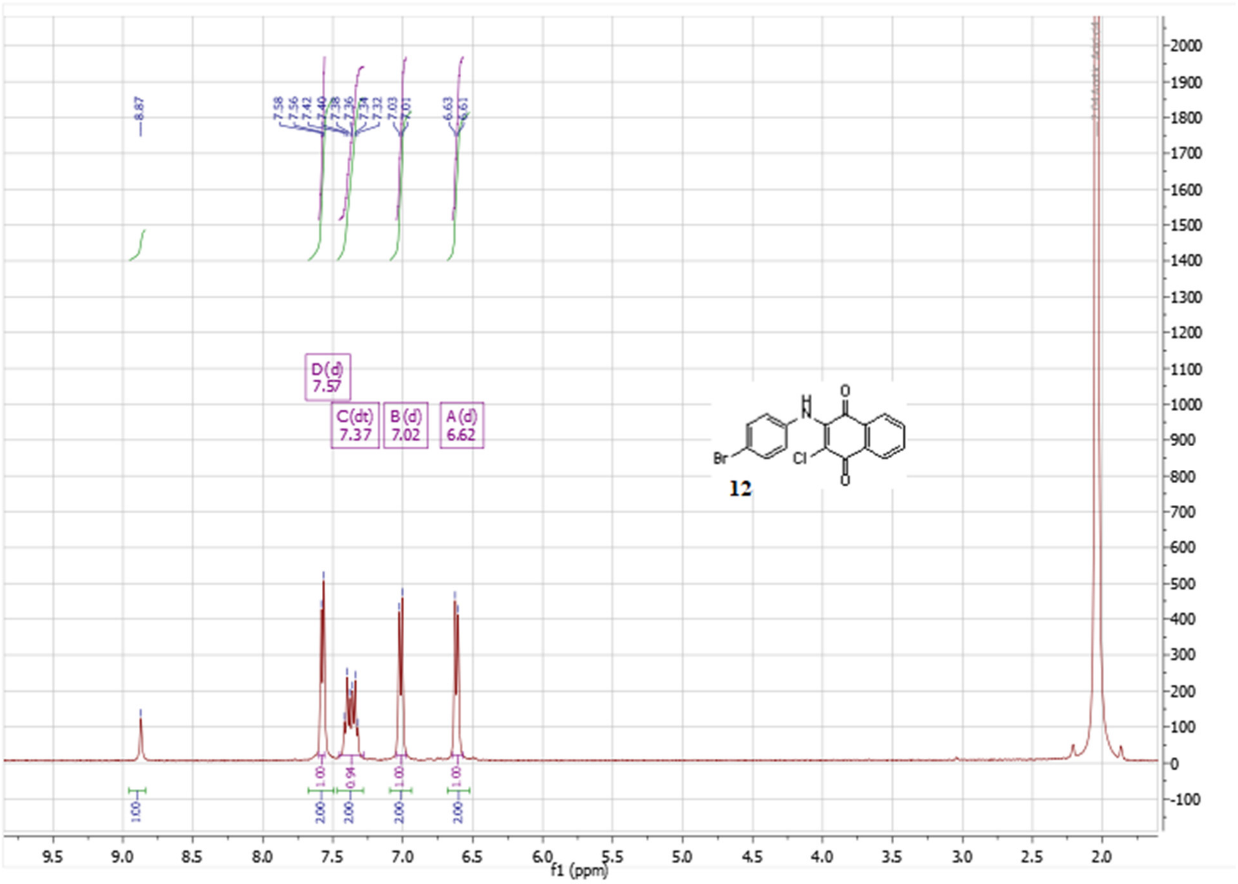

Figure S7: ^{1}H-NMR spectrum of compound **12**.

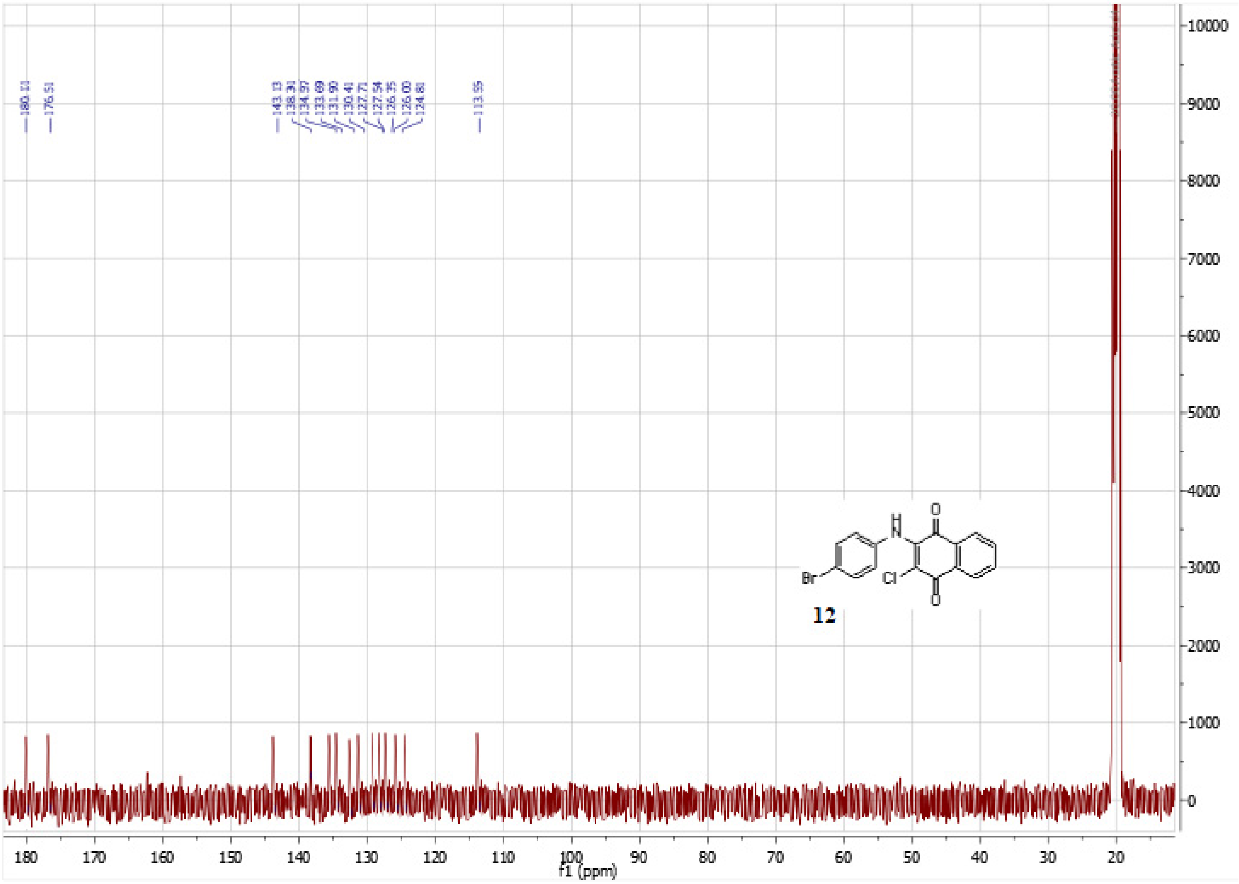

Figure S8: ^{13}C-NMR spectrum of compound **12**.

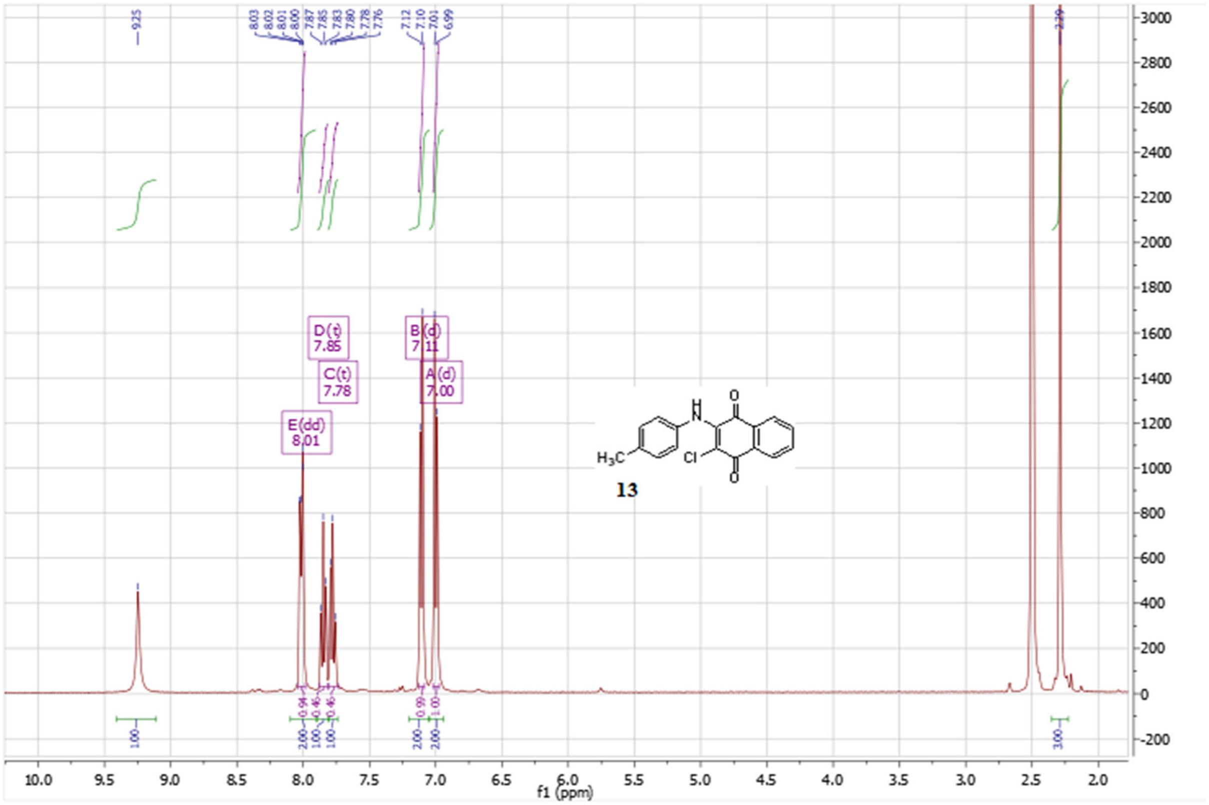

Figure S9: ^{1}H-NMR spectrum of compound **13**.

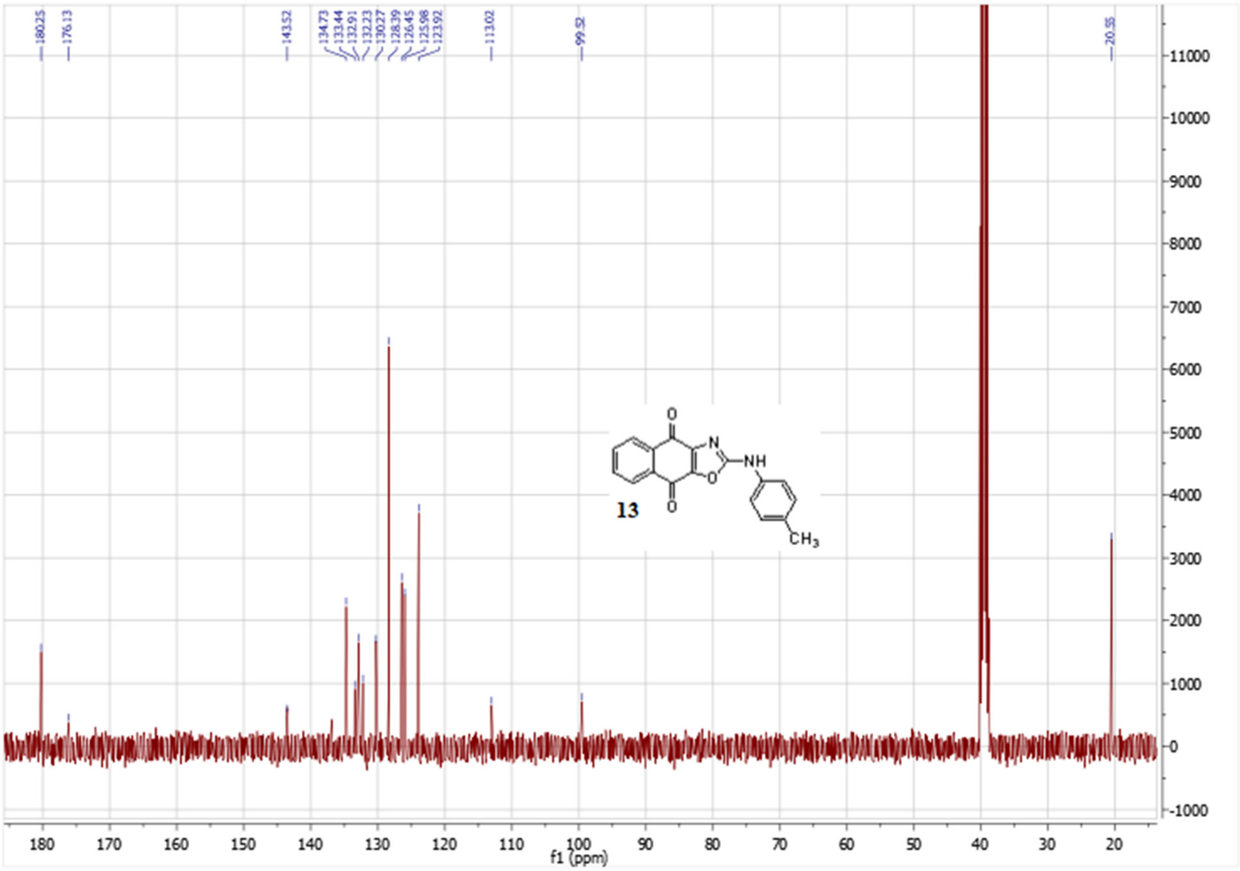

Figure S10: ^{13}C-NMR spectrum of compound **13**.

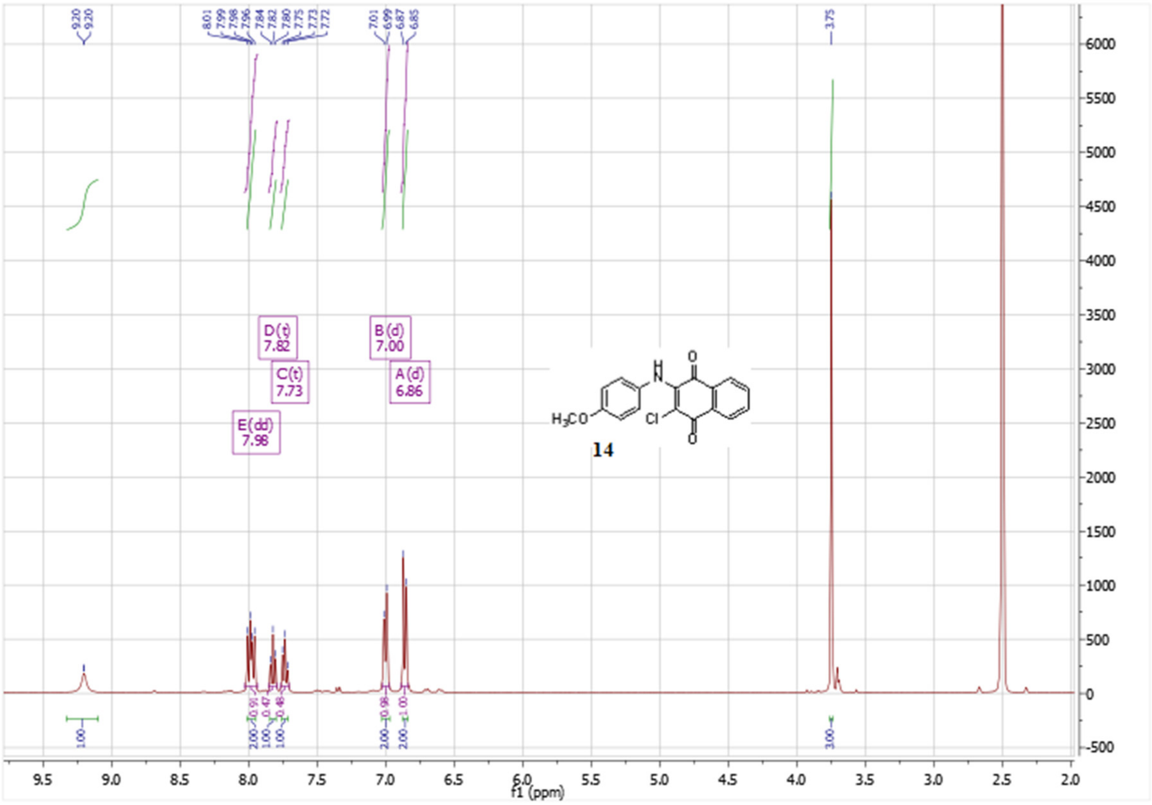

Figure S11: ^{1}H-NMR spectrum of compound **14**.

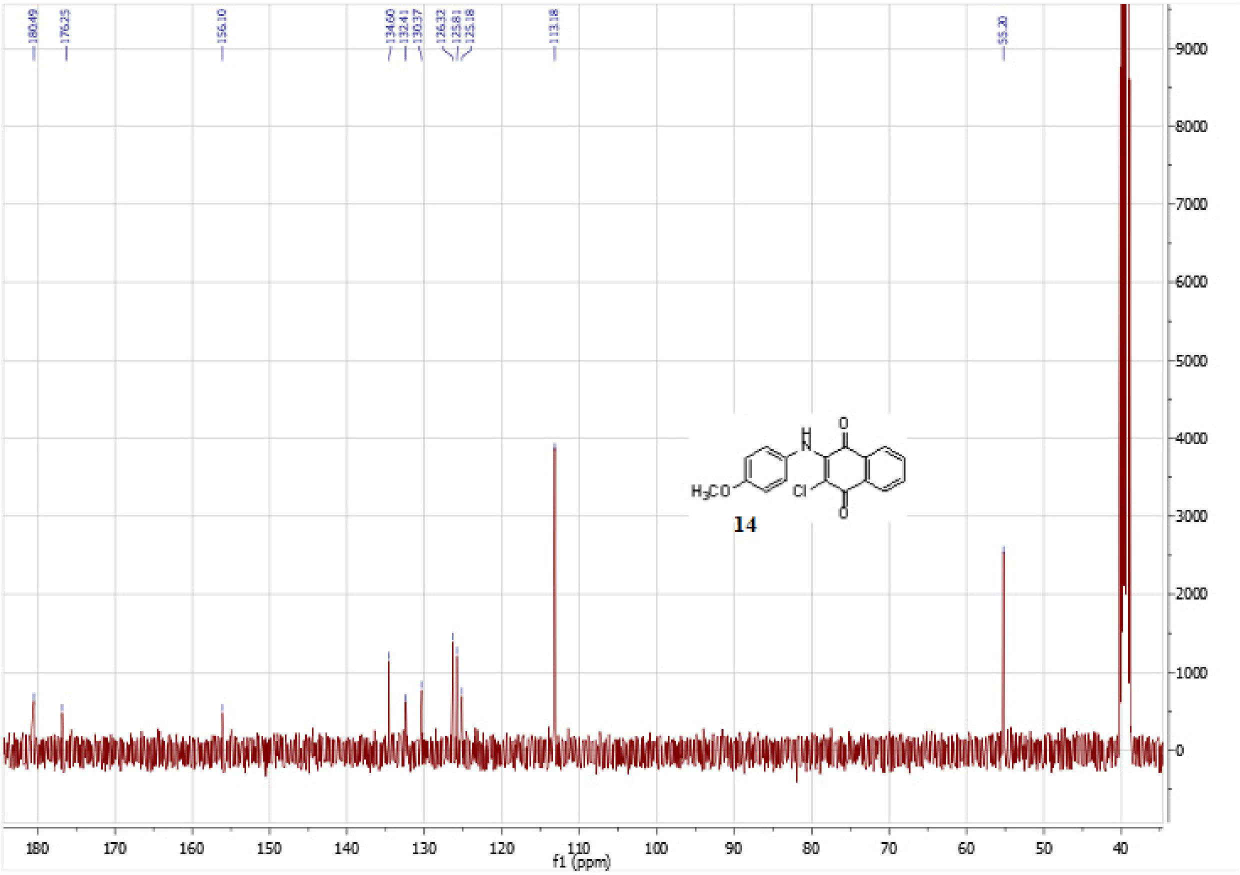

Figure S12: ^{13}C-NMR spectrum of compound **14**.

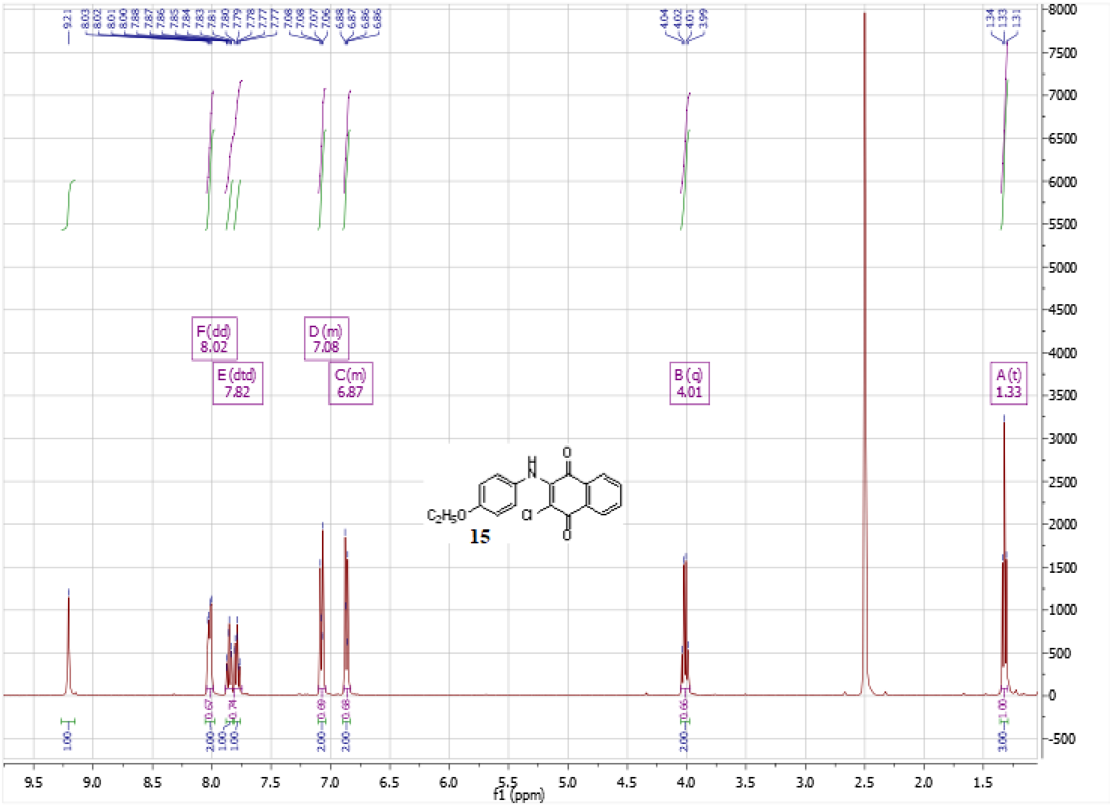

Figure S13: ^{1}H-NMR spectrum of compound **15**.

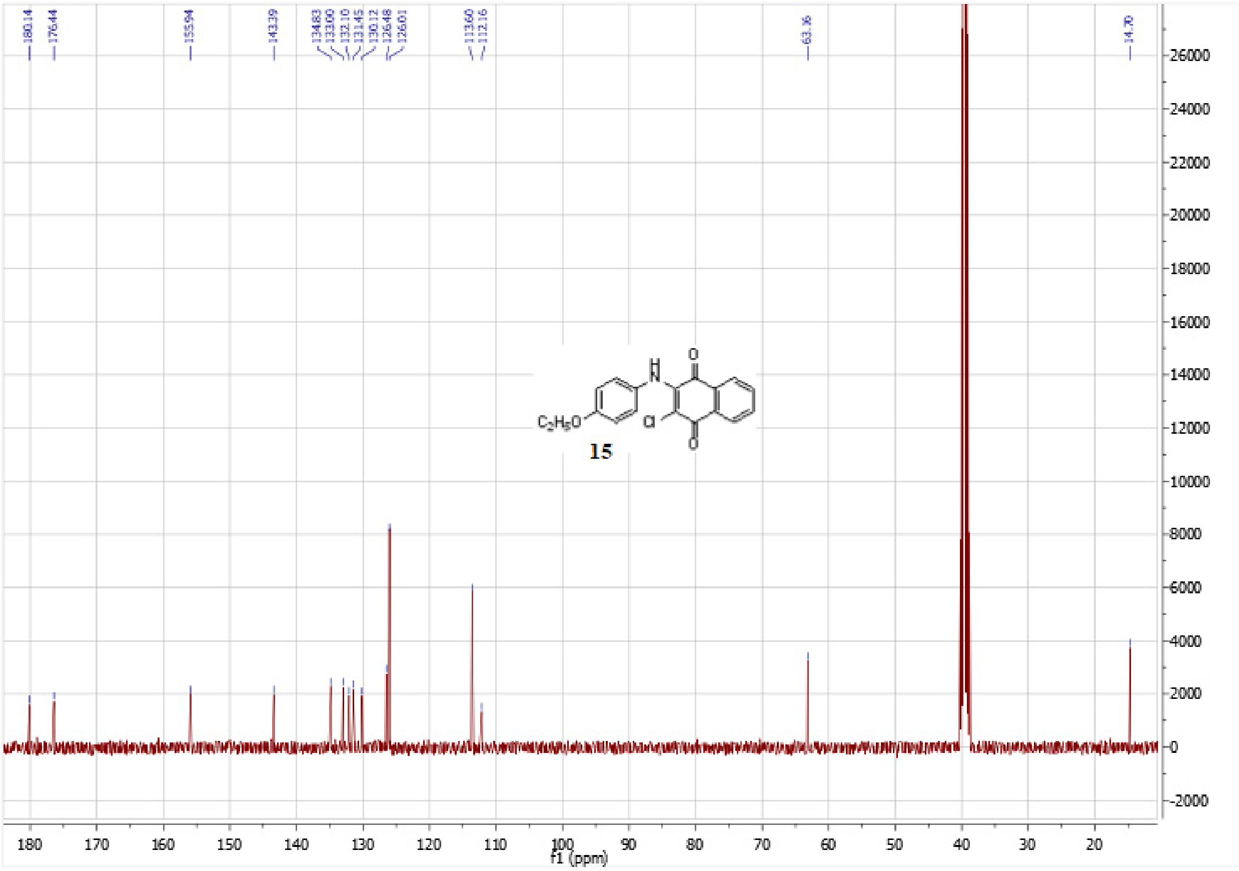

Figure S14: ^{13}C-NMR spectrum of compound **15**.

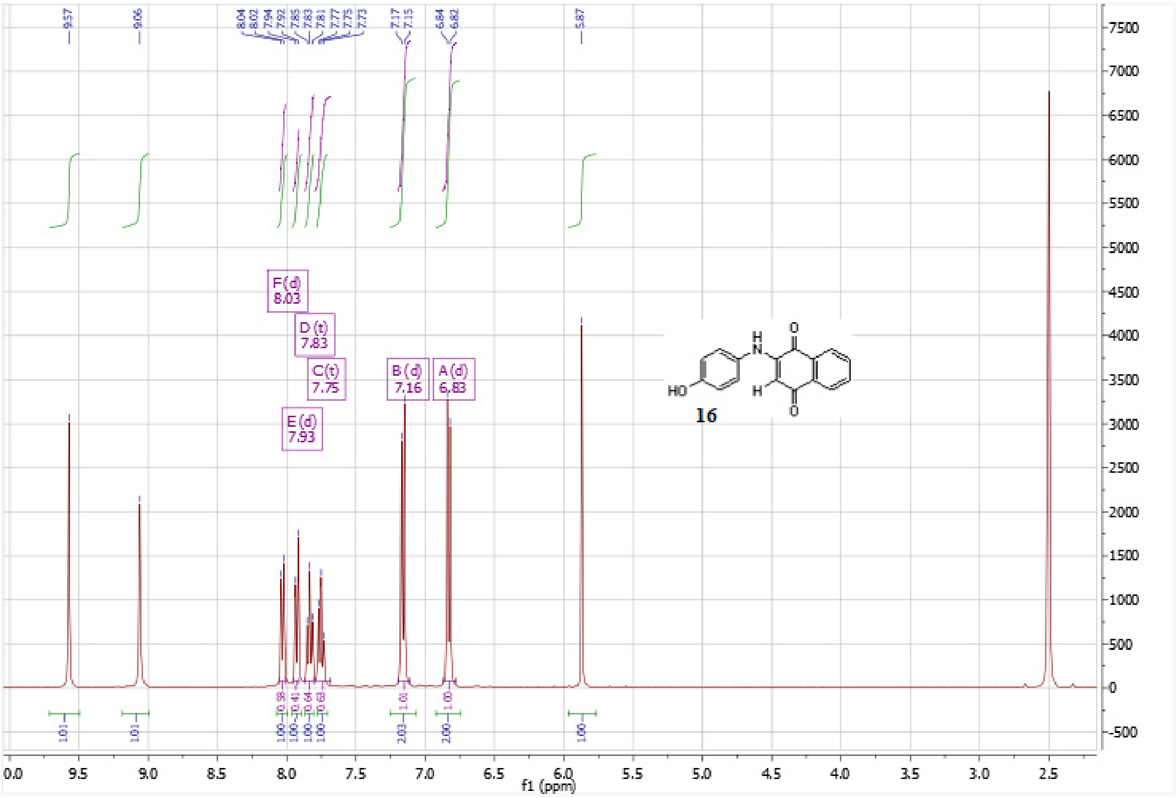

Figure S15: ^{1}H-NMR spectrum of compound **16**.

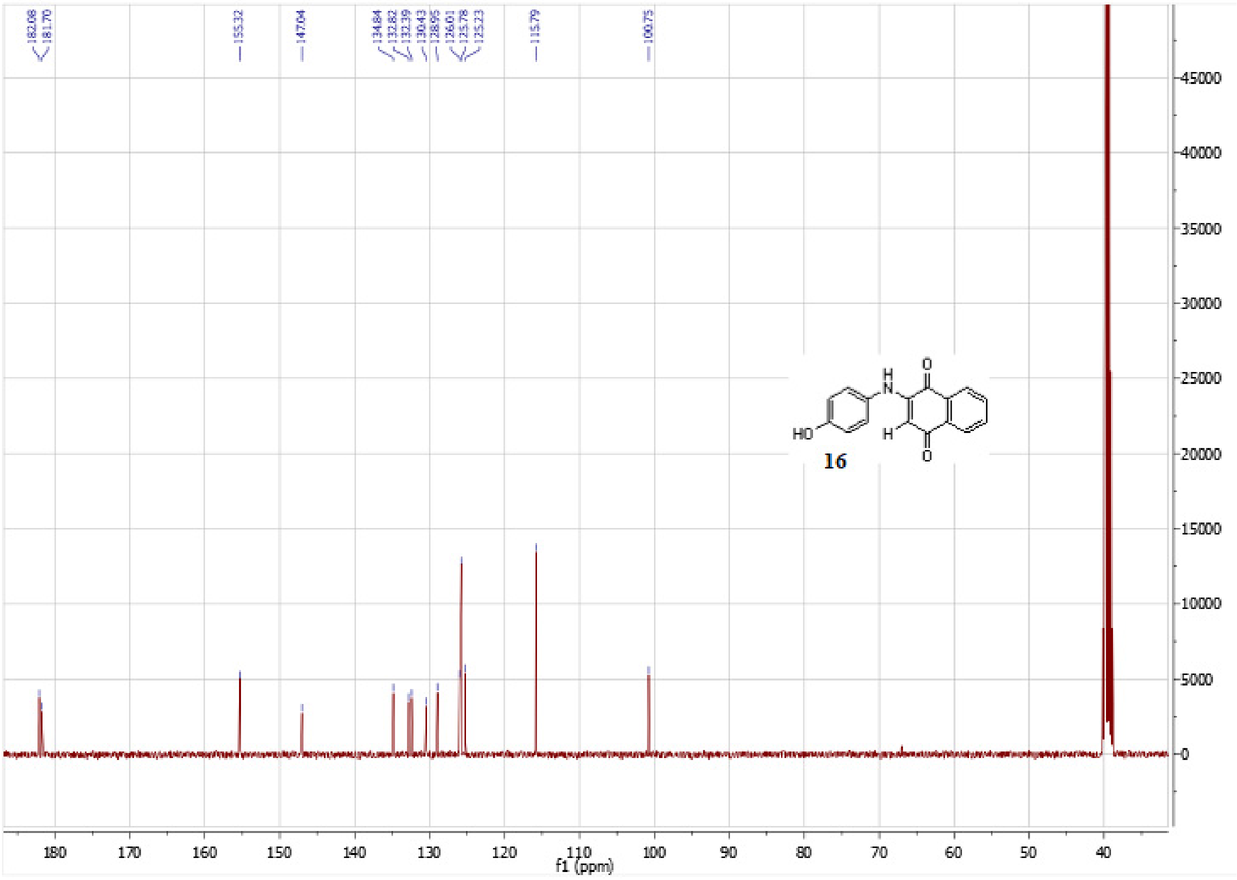

Figure S16: ^{13}C-NMR spectrum of compound **16**.
